# FGFR4 promotes CAF activation through the CXCL10-CXCR3 axis in colon cancer

**DOI:** 10.1038/s41419-025-07588-y

**Published:** 2025-05-30

**Authors:** Eun-Gene Sun, Ji-Na Choi, Mi-Ra Park, Dae-Hwan Kim, MinJeong Sung, Hyun-Jeong Shim, Jun-Eul Hwang, Woo-Kyun Bae, Chaeyong Jung, Young-Kook Kim, Ik-Joo Chung, Sang-Hee Cho

**Affiliations:** 1 Department of Internal Medicine, Division of Hematology and Oncology, Chonnam National University Medical School and Hwasun Hospital, Hwasun, Jeollanam-do Republic of Korea; 2 National Immunotherapy Innovation Center, Chonnam National University Medical School, Hwasun, Jeollanam-do Republic of Korea; 3 Combinatorial Tumor Immunotherapy MRC Center, Chonnam National University Medical School, Hwasun, Jeollanam-do Republic of Korea; 4 Department of Anatomy, Chonnam National University Medical School, Hwasun, Jeollanam-do Republic of Korea; 5 Department of Biochemistry, Chonnam National University Medical School, Hwasun, Jeollanam-do Republic of Korea

**Keywords:** Cancer microenvironment, Cancer microenvironment

## Abstract

Cancer-associated fibroblasts (CAFs) promote the malignant phenotype of cancer through crosstalk with tumor and immune cells within the tumor microenvironment. Therefore, the mechanisms underlying CAF activation require in-depth study to develop strategies targeting CAFs during cancer immunotherapy. In this study, we investigated the role of FGFR4 in CAF regulation in colon cancer. FGFR4-overexpressing cancer cells promoted CAF abundance and activation in vivo, while also inducing the differentiation of normal fibroblasts into CAFs via their secretome. Mechanistically, FGFR4 induced CXC-chemokine ligand (CXCL) 10 production by upregulating Toll-like receptor 3-interferon regulatory factor-interferon beta (IFNβ) signaling and the autocrine action of IFNβ. CXCL10 increased CAF marker expression in fibroblasts, including alpha-smooth muscle actin and vimentin. CXCL10 also promoted CAF migration, invasion, and contractibility, which reflects CAF activation. In contrast, knocking down CXCL10 or neutralizing antibodies abolished CAF marker expression in fibroblasts. Inhibition of CXC receptor type (CXCR) 3, the cognate receptor of CXCL10, also impaired CAF function. In human colon cancer samples, *FGFR4* and *CXCL10* expression was positively correlated with CAF marker expression. Finally, dual inhibition of FGFR4 and CXCR3 suppressed tumor growth, accompanied by CAF downregulation. Our findings reveal the mechanism through which FGFR4 promotes CAF differentiation/activation in TME via the CXCL10-CXCR3 axis, highlighting the potential of co-targeting FGFR4 and CXCR3 as a therapeutic strategy for patients with stromal-dominant tumors.

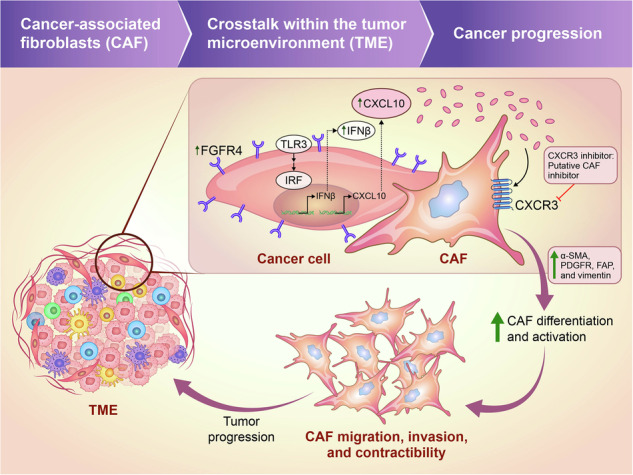

## Introduction

Tumor cells do not exist in isolation in vivo; rather, they exist within a supporting system known as the tumor microenvironment (TME). Tumor progression depends on TME remodeling via crosstalk between tumor cells and TME components, such as immune cells, stromal cells, and the extracellular matrix. Thus, interrupting the vicious tumor–TME crosstalk is an attractive approach for targeted cancer therapy [1].

Among the TME components, abundant stroma and fibrosis are critical for conferring an aggressive cancer phenotype. Cancer-associated fibroblasts (CAFs) are the major components of stromal cells and can originate from normal tissue-resident fibroblasts or non-fibroblastic mesenchymal cells, which are induced by tumor cells via multiple mechanisms. The several key factors contributing to CAF differentiation or activation include transforming growth factor-beta (TGF-β), platelet-derived growth factor (PDGF), fibroblast growth factor (FGF), interleukin-1 (IL)-1β, CXC-chemokine ligand (CXCL)12/stromal cell-derived factor 1, and CC chemokines, which are produced by both tumor and stromal cells [2]. CAFs also secrete growth factors, such as TGF-β and PDGF, and promote tumor progression through the autocrine or paracrine repertoire [3]. Several CAF-derived chemokines such as CCL2, CCL26, IL6, CXCL1, and CXCL8 mediate tumor progression, angiogenesis [4], and drug resistance [5–7], suggesting that tumor–CAF crosstalk is a bidirectional, dynamic, and adaptive process. The evolving spatiotemporal interaction between tumor cells and CAFs triggers a cascade of events that results in tumor progression and poor treatment outcomes.

The FGF/FGF receptor (FGFR) tyrosine kinase signaling pathway plays a crucial role in embryonic development, tissue repair, and tumor angiogenesis and progression through multiple mechanisms [8]. Among FGFR family members (FGFR1–4), FGFR4 is a candidate key factor in hepatocellular and thyroid carcinoma [9, 10]. FGFR4 promotes tumor progression by binding to its ligands, including FGF19, and upregulates downstream signaling cascades such as Ras-Raf-MAPK and PI3K-AKT to regulate cell growth, differentiation, and survival, together with cell migration by inducing epithelial–mesenchymal transition (EMT) [11]. Several FGFR4-selective inhibitors were recently used to treat solid tumors but showed limited efficacy [12, 13]. Tao et al. suggested that this reduced efficacy involved chemoresistance of the FGFR4 inhibitor, which is associated with FGFR redundancy because of the interconnection between FGFRs [14]. In addition, FGFR4 can affect the TME, particularly CAFs; however, crosstalk between tumor–TME network functions in the context of the FGFR4 signaling pathway remains unclear.

We previously reported the role of FGFR4 in EMT induction and the mechanism underlying anti-EGFR chemoresistance based on amphiregulin secretion in colon cancer [15, 16]. However, whether FGFR4-overexpressing tumors change TME, via its secretome, particularly CAFs, is unclear. In this study, we explored whether FGFR4 signaling induces CAF differentiation and the underlying mechanism to provide new insights for exploring CAF-targeted therapies in colon cancer.

## Materials and methods

### Primary tumor-derived CAF isolation

After tumor isolation from a mouse subcutaneous tumor model, tumor tissues were dissociated and homogenized using a gentle MACS dissociator (Miltenyi Biotec, Bergisch Gladbach, Germany) with a mouse Tumor Dissociation Kit (Miltenyi Biotec; 130-096-730). After filtering the samples through a 40-µm strainer, we isolated fibroblasts from the cell suspension using a Tumor-Associated Fibroblast Isolation Kit (Miltenyi Biotec; 130-116-474). The isolated cells were cultured in Dulbecco’s modified Eagle’s medium/F12 (Gibco, Grand Island, NY, USA) supplemented with 10% fetal bovine serum (FBS) (Gibco) at 37 °C and 5% CO_2_. The medium was replaced after 24 h. CAFs were identified based on their morphology and assessed for gene expression of CAF markers.

### Cell culture

The CT-26, HT-29, and NIH/3T3 cell lines were obtained from the American Type Culture Collection (Manassas, VA, USA) and Korean Cell Line Bank (Seoul, Korea). The cells were cultured in Dulbecco’s modified Eagle’s medium or Roswell Park Memorial Institute-1640 medium containing 10% FBS, 100 U/mL penicillin, and 100 μg/mL streptomycin (Invitrogen, Carlsbad, CA, USA) at 37 °C and 5% CO_2_.

### Preparation of conditioned medium

The cells were grown to confluence, and the culture medium was replaced with a serum-free medium. After 48 h of culture, the conditioned medium (CM) was filtered using a 0.2-μL filter and centrifuged at 300 × *g* for 10 min. After removing cell debris, the supernatant was collected as CM.

### Plasmid construction

DNA fragments of mouse *Fgfr4* and human *FGFR4* were PCR-amplified and cloned into a pcDNA6/Myc-His A vector (Invitrogen) using an In-Fusion HD Cloning kit (Clontech Mountain View, CA, USA). The primers used for cloning are listed in Table S1. All constructs were validated using DNA sequencing.

### Reagents

Mouse CXCL10 (R&D Systems, Minneapolis, MN, USA; 466-CR) and mouse TGFβ (R&D Systems; 7666-MB) were used as recombinant proteins. The following inhibitors were used: BLU9931 (Merck, Kenilworth, NJ, USA; 538776), AMG487 (MedChemExpress, Monmouth Junction, NJ, USA; HY-15319), TAK779 (MedChemExpress; HY-13406), NBI74330 (MedChemExpress; HY-15320), and SCH546738 (MedChemExpress; HY-10017).

### Cell proliferation, migration, and invasion assay

Cell proliferation was evaluated using Cell Counting Kit-8 (CCK-8 kit, Sigma-Aldrich, St. Louis, USA). Cell migration and invasion were measured using a Transwell chamber system (Corning Life Science, Corning, NY, USA). A cell culture medium containing 5% FBS was added to the bottom chamber. Cells were seeded onto the top of a non-coated and Matrigel-coated (1 µg/mL) 24-well Transwell filter chamber for migration and invasion assays, respectively. After 16–24 h, the cells were stained with Diff-Quick solution (Sysmex, Kobe, Japan). The area of migrated or invaded cells was calculated using ImageJ (National Institutes of Health, Bethesda, MD, USA).

### Collagen gel contraction assay

For the collagen gel contraction assay, isolated CAF or NIH/3T3 fibroblasts were adjusted to 1.5 × 10^5^ cells/mL in a cell culture medium. The cell suspension (800 µL) was pipetted into a 1.5-mL tube, and 400 µL of 3 mg/mL rat tail collagen I solution (A1048301, Thermo Fisher Scientific, Waltham, MA, USA) was added to the cell suspension and mixed by pipetting. The cell–collagen mixture was immediately transferred to a well and the gels were allowed to solidify for 1 h at 37 °C. Cell culture medium was added to each well, and the plates were incubated at 37 °C. The collagen gel was imaged, and the gel area was calculated according to the gel diameter. The percentage of gel area to the initial gel area was compared between the different groups.

### RNA sequencing analysis

Total RNA (1 µg) was extracted from Fgfr4-overexpressing CT-26 and empty vector (EV) control CT-26 cells using a Ribospin RNA extraction kit (GeneAll Biotechnology, Seoul, Korea). After quality evaluation, libraries were generated using a TruSeq RNA Library Prep Kit V2 and sequenced on an Illumina platform (San Diego, CA, USA). LAS, Inc. (Seoul, Korea) carried out all sequencing analyses. RSEM with STAR aligner was used to map the mRNA-sequencing (mRNA-seq) data to the mouse reference genome (version mm10). To identify differentially expressed genes, we selected genes with an average of at least one for each normalized read. Genes with a P*-*value of 0.001 or less and an absolute log2 fold-change value of 2 were considered differentially expressed compared to the reference genome. We identified 96 significantly upregulated and 44 significantly downregulated genes. Upregulated genes were subjected to Reactome pathway analysis using MSigDB [17].

### Real-time quantitative PCR

RNA isolation and cDNA synthesis were conducted using a Ribospin RNA extraction kit (214–150, Korea) and GoScript system (Promega, Madison, WI, USA), respectively. Reverse transcription-quantitative PCR (RT-qPCR) was performed using the CFX96 Real-Time PCR Detection System (Bio-Rad Laboratories, Hercules, CA, USA). Primers used are listed in Table S2. *GAPDH* and *18S rRNA* were used as internal controls for expression normalization. Fold changes in gene expression were calculated using the ΔΔCT method [18].

### Western blotting

Cells were lysed using a protein extraction reagent (78501; Thermo Fisher Scientific). Equal amounts of whole-cell lysates were resolved using SDS-PAGE and transferred onto polyvinylidene fluoride membranes (IPVH00010, Millipore, Billerica, MA, USA). Membranes were blocked using the SuperBlock^TM^ T20 blocking buffer (37515; Thermo Fisher Scientific) and incubated overnight at 4 °C with primary antibodies (antibodies listed in Table S3). The membranes were probed with horseradish peroxidase-conjugated secondary antibodies for 1 h at 25 °C and visualized using a low-light imaging system (LAS-4000 mini; Fujifilm, Tokyo, Japan). Band intensities were quantified using the Multi-Gauge software (version 3.0; Fujifilm).

### Enzyme-linked immunosorbent assay

Cytokine and chemokine levels in CM were measured using commercial enzyme-linked immunosorbent assay (ELISA) kits for mouse IFN-β (DY8234-05) and CXCL10 (DY466-05) (BD Biosciences, San Jose, CA, USA).

### Immunofluorescence assay

Isolated tumor-derived CAFs were fixed with 4% (v/v) paraformaldehyde for 10 min and washed with PBS. The cells were permeabilized with 0.1% (v/v) Triton-X100 for 10 min. The primary and fluorescence-conjugated secondary antibodies are listed in Table S3. Images were captured using a fluorescence microscope (Zeiss, Jena, Germany).

### Clinical cancer specimens from human patients

Biospecimens of colon cancer and matched normal tissues were provided by the Biobank of Chonnam National University Hwasun Hospital Institutional Review Board (approval number: IRB CNUHH-2018-173). Stage II or III colon cancer (n = 137) and matched normal tissue samples (n = 137) were used to examine *FGFR4* and CAF marker expression. Pearson’s correlation analyses were performed using GraphPad Prism version 10 (GraphPad, Inc., La Jolla, CA, USA), using the normalized gene expression values.

### Animal models and ethics approval

Eight-week-old male BALB/C mice were obtained from Orient Bio, Inc. (Seongnam, Korea) and maintained in a pathogen-free facility to establish a syngenic tumor model. In total, 1 × 10^6^ CT-26/EV or CT-26/FGFR4 cells were subcutaneously injected into the dorsal flanks of mice (n = 5/group) to assess the effects of FGFR4 overexpression on tumor growth and CAF activation. Tumor size was measured with calipers and tumor volume was calculated using the formula: Tumor Volume = 0.5 × Length × Width^2^.

To examine the effect of FGFR4 and CXCR3 inhibitors on tumor growth, 3 × 10^5^ CT-26/FGFR4 cells were subcutaneously injected. When the tumor volume reached ~70 mm^3^, mice were randomly allocated to the vehicle, BLU9931, AMG487, and combination groups. BLU9931, as an FGFR4 inhibitor, was formulated in 0.5% carboxymethylcellulose/1% Tween80, and given to mice at 30 mg/kg orally twice daily [12]. AMG487 was dissolved in 20% hydroxypropyl-β-cyclodextrin (HP βCD; Sigma-Aldrich) as previously described and mice were treated twice daily on days with s.c. injections of 5 mg/kg of AMG487 [19]. Mice were monitored daily and the tumor sample was collected after sacrifice. The tumor sections were stained with hematoxylin and eosin, α-smooth muscle actin (SMA), Ki-67, CD8, CD86, and CD206 (T&P Bio, Gyeonggi, Korea). The slides were imaged using a Zeiss Axio Scan.Z1 slide scanner (Carl Zeiss). ImageJ software was used to score Immunohistochemistry (IHC)-stained sections. All animal care procedures and experiments were performed according to the Institutional Animal Care and Use Committee (IACUC) guidelines. This study was approved by the IACUC of Chonnam National University Medical School (Approval No. CNU IACUC-H-2023-6).

### Toxicity assay

The mouse body weight was monitored throughout the in vivo experiment. After scarifying the mice, whole blood samples were collected in Microtainer EDTA tubes (BD#365974). The hemogram (complete blood cell count) was analyzed in the Laboratory Animal Resource Center of the Gwangju Institute of Science and Technology (GIST LARC) using an Exigo H400 Veterinary Hematology Analyzer (Boule Medical AB, Sweden). Blood urea nitrogen (BUN), alanine aminotransferase (ALT), and aspartate aminotransferase (AST) levels were measured using a blood biochemistry analyzer (Catalyst One, IDEXX Laboratories, Inc., Westbrook, ME, USA).

### Statistical analysis

Pearson’s and Spearman’s correlation analyses were performed to measure correlations between two factors in samples from cancer patients. The correlation was considered significant at P < 0.05 and Pearson’s R > 0.30. Data are presented as mean ± SD for continuous variables and as frequencies and proportions for categorical variables. Data were compared using Student’s *t*-test (comparing two variables) or one-way analysis of variance (comparing multiple variables). Statistical significance was considered at P < 0.05. All experiments were performed in triplicate unless otherwise noted. GraphPad Prism version 10 was used for all statistical tests and plot generation.

## Results

### FGFR4 promotes tumor growth and CAF differentiation/activation in the TME

**Fig. 1:**
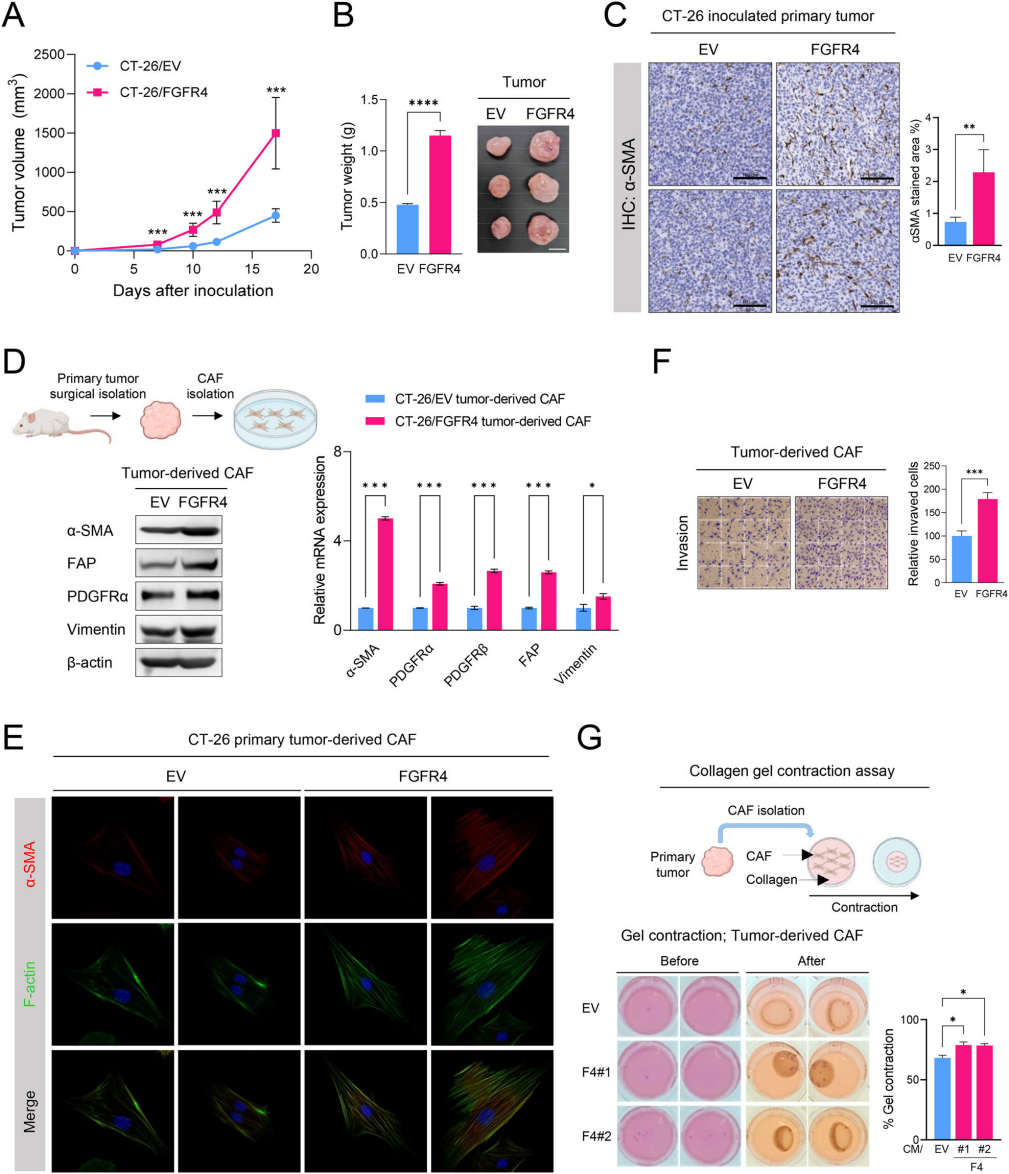
FGFR4 promotes tumor growth and CAF activation in vivo. Syngeneic BALB/c mice were subcutaneously injected with CT-26/EV- or CT-26/FGFR4-overexpressing cells (n = 5/group). CAFs were isolated from the primary tumor at the end of the study (D–G). **A** Average tumor growth curves after inoculation. **B** Comparison of tumor tissue weight at the end of the study. **C** Representative IHC images and quantification of α-SMA staining in primary tumor from CT-26/EV or FGFR4 groups. **D** Expression of CAF markers in isolated CT-26/EV or CT-26/FGFR4 tumor-derived CAFs determined using western blot analysis (left panel) and RT-qPCR (right panel). β-actin and *Gapdh* expression were used as an internal reference control in western blotting and RT-qPCR analyses, respectively. **E** Immunofluorescence assay for α-SMA and F-actin in tumor-derived CAFs. Comparison of the invasion potential (**F**) and contractility (**G**) of tumor-derived CAFs. Data are presented as mean ± SD from three independent experiments. Statistical significance: *P < 0.05, ***P < 0.001, and ****P < 0.0001 using Student’s *t*-test and one-way ANOVA. EV empty vector, F4 FGFR4.

To investigate the impact of FGFR4 overexpression on tumor growth and CAF activation, we established a subcutaneous tumor model using mouse colon cancer CT-26/EV or CT-26/FGFR4 stable-expressing cell lines. The xenografted mice with CT-26/FGFR4 showed a marked increase in tumor growth and weight. The number of α-SMA-positive cells significantly increased in TME, suggesting that FGFR4 overexpression in cancer cells enhances CAF abundance in TME (Fig. 1A–C).

We further explored the role of FGFR4 in CAF activation within TME. We assessed the expression of CAF markers and their activation status in CAFs derived from either CT-26/EV or CT-26/FGFR4 tumors. Compared with those in CT-26/EV cells, CT-26/FGFR4 tumor-derived CAFs exhibited higher expression levels of CAF markers, such as α-SMA, fibroblast activation protein (FAP), PDGF receptor (PDGFR), and vimentin, at both mRNA and protein levels (Fig. 1D). Immunofluorescence assays confirmed that CT-26/FGFR4-derived CAFs showed elevated α-SMA expression and a distinct elongated spindle-like morphology (Fig. 1E). The activation status of these CAFs was validated through invasion and collagen gel contraction assays, which revealed increased invasiveness and contractility (Fig. 1F, G). These results indicate that FGFR4 promotes tumor progression, concomitant with CAF activation in TME.

### FGFR4 induces CAF differentiation and activation via the secretome

**Fig. 2:**
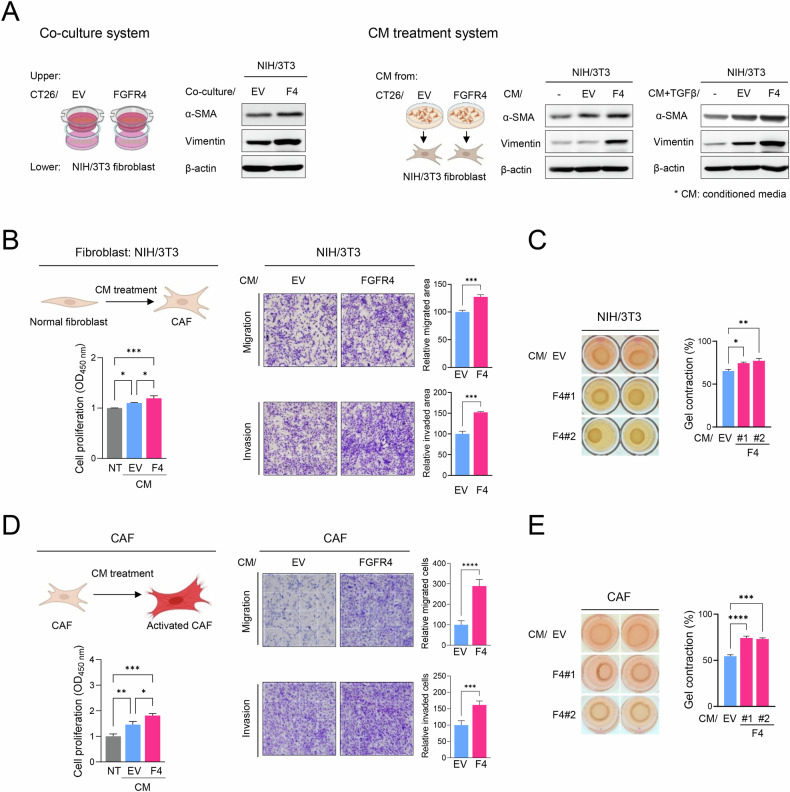
FGFR4 induces CAF differentiation and activation via its secretome. **A** NIH/3T3 fibroblasts were cocultured with either CT-26 stable cells or their CM, and CAF marker expression was determined via western blotting analysis. **B** CCK-8 assay results of NIH/3T3 proliferation after CM treatment for 48 h (left panel) and the migration and invasion of NIH/3T3 cells incubated with CM obtained from CT-26/EV or CT-26/FGFR4-overexpressing cells (right panel). **C** Gel contraction assay for NIH/3T3 cells incubated with CM. **D** CCK-8 assay results for the proliferation of CM-treated CAF (left panel). Migration and invasion assay of CAFs incubated with CM (right panel). **E** Gel contraction assay for CAFs incubated with CM. Data are presented as mean ± SD from three independent experiments. Statistical significance: *P < 0.05, **P < 0.01, ***P < 0.001; ****P < 0.0001 using Student’s *t*-test and one-way ANOVA. EV empty vector, F4 FGFR4.

To investigate the influence of the cancer secretome on normal fibroblast differentiation into CAFs, we cocultured NIH/3T3 fibroblasts with stable cancer cells or their CM and then examined CAF marker expression via western blotting to assess CAF differentiation. In line with in vivo findings, in both coculture and CM treatment systems, *Fgfr4*-overexpressing cancer cells strongly induced the expression of CAF markers such as *α-SMA* and *vimentin* in NIH/3T3 cells through their secretome, suggesting that the *Fgfr4*-overexpressing cancer-derived secretome is involved in CAF differentiation (Fig. 2A). Moreover, compared with CM from CT-26/EV cells, the CM from CT-26/FGFR4 cells significantly enhanced the proliferation, migration, invasion, and contractility of NIH/3T3 cells, indicating that CAF differentiation was promoted in resting fibroblasts (Fig. 2B, C). To further examine the effect of the cancer secretome on CAF activation, we isolated CAFs from CT-26-inoculated tumors, cultured these cells with CM from CT-26 stable cancer cells, and assessed CAF activities [20–22]. Compared with CT-26/EV-derived CM, CM from CT-26/FGFR4 cells significantly enhanced CAF activation. These findings support the contribution of FGFR4 to CAF differentiation and activation through its secretome.

### Transcriptome analysis of Fgfr4-overexpressing colon cancer cells

**Fig. 3:**
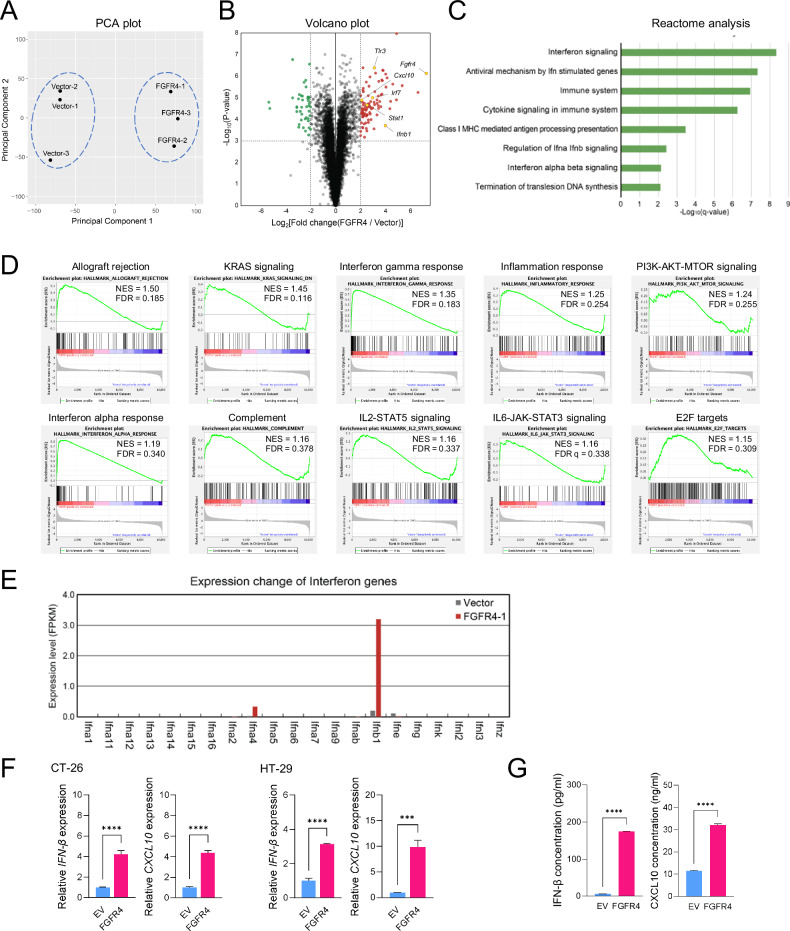
Transcriptome analysis of FGFR4-overexpressing colon cancer cells. **A** Principal Component Analysis (PCA) plot showing distinct separation of EV and FGFR4 overexpression CT-26 groups (n = 3/group). **B** Volcano plot of DEGs in FGFR4-overexpressing CT-26 cells compared with those in EV controls. DEGs were defined as genes with a P < 0.001 and an absolute value of >2 for the log2 of fold-change in FGFR4-overexpressing cells compared with that in controls. Green and red dots show significantly downregulated and upregulated DEGs, respectively. **C** Reactome analysis for the 96 significantly upregulated genes. **D** Enrichment plots for the top ten gene sets enriched in Gene Set Enrichment Analysis (GSEA) Hallmark analysis. These plots show the running enrichment score (ES) profile and the distribution of gene set members along the rank-ordered list of genes. In each graph, the probes on the far left (red) represent genes most correlated with upregulation, while those on the far right (blue) indicate genes most correlated with downregulation in the FGFR4-overexpressing group. The green line represents the running ES across the gene list. The normalized enrichment score (NES) and false discovery rate (FDR) are provided for each gene set. **E** Changes in expression of IFN genes based on RNA-seq data. **F** RT-qPCR analysis confirmed that FGFR4 overexpression led to increased expression of *Ifnβ* and *Cxcl10* in CT-26 and HT-29 cells. *Gapdh* was used as an internal reference control gene. **G** ELISA analysis of secretory Ifnβ and Cxcl10 levels in conditioned media of CT-26/EV and CT-26/FGFR4 cells. Data are presented as mean ± SD from three independent experiments. Statistical significance: ***P < 0.001, ****P < 0.0001 using Student’s *t*-test. DEGs, differentially expressed genes.

Principal component analysis (PCA) plot of RNA-seq data showed clear segregation between the CT-26/FGFR4 and CT-26/EV control cells, indicating distinct gene expression profiles resulting from FGFR4 overexpression (Fig. 3A). RNA-seq analysis of CT-26/EV and CT-26/FGFR4 cells revealed 96 upregulated and 44 downregulated genes in Fgfr4-overexpressing cells compared with those in EV controls (fold-change > 2, P < 0.001) (Fig. 3B). Reactome pathway analysis revealed that upregulated genes were associated with interferon (IFN) signaling, antiviral mechanisms involving IFN-stimulated genes, the immune system, and cytokine signaling (Fig. 3C). Gene Set Enrichment Analysis (GSEA) analysis also demonstrated enrichment of immune-related pathways, such as interferon alpha response, interferon-gamma response, and cytokine signaling, indicating a robust activation of immune and interferon signaling pathways due to FGFR4 overexpression (Fig. 3D). Notably, *Ifnβ1* and its downstream target gene, IFN-inducible protein 10 (*IP-10* and *Cxcl10*), were specifically upregulated by FGFR4 overexpression (Fig. 3E, F, and Fig. S1). *Ifnβ* and *Cxcl10* encode secretory molecules associated with the TLR3-TBK-IRF and IFN-signal transducer and activator of transcription (STAT)-CXCL10 signaling pathways, which overlap with the upregulated pathways identified in Reactome and GSEA analysis. We validated the increased production of IFN-β and CXCL10 under FGFR4 overexpression using ELISA analysis (Fig. 3G).

### FGFR4 induces CXCL10 expression via TLR3-IRF-IFNβ axis activation in colon cancer

**Fig. 4:**
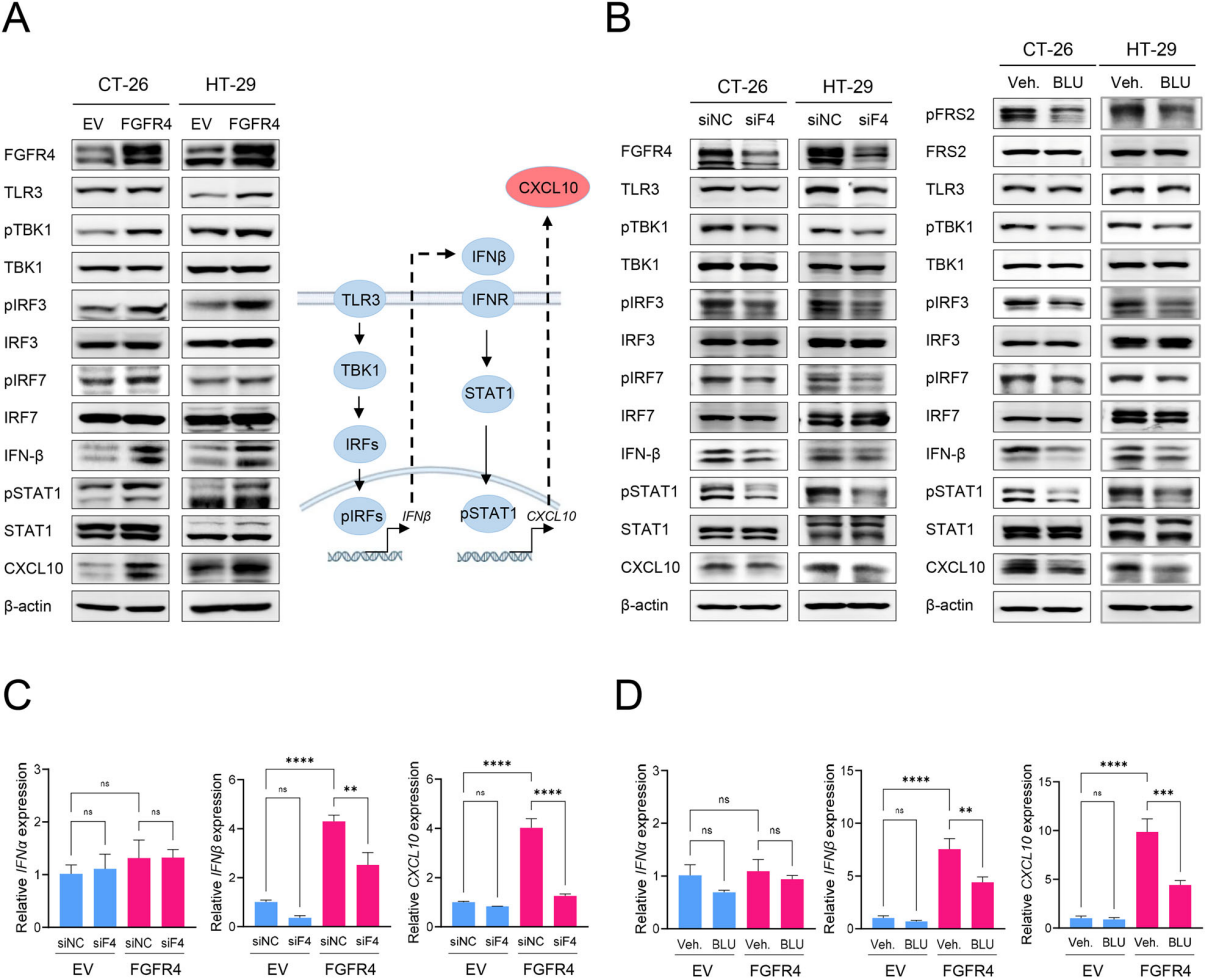
FGFR4 induces CXCL10 secretion via activation of the TLR3-TBK-IRF axis and autocrine action of IFNβ. **A** Left: FGFR4-mediated activation of the TLR3-TBK-IRF and IFN signaling pathways was determined using western blotting for the indicated protein markers in CT-26 and HT-29 cells. Phosphorylated proteins are denoted by “P” before the protein name. Right: Schematic representation of the TLR3-TBK-IRF and IFN-STAT-CXCL10 signaling pathway. **B** The TLR3-TBK-IRF and IFN-STAT-CXCL10 signaling pathways are suppressed following FGFR4 knockdown or pharmacological inhibition by BLU9931 in FGFR4-overexpressing CT-26 and HT-29 cells. Cells were transfected with siRNA targeting FGFR4 for 48 h or treated with 1 μM BLU-9931 for 24 h. Western blot analysis shows a reduction in TBK1, IRF, and STAT1 phosphorylation, and the downstream targets IFN-β and CXCL10 following FGFR4 knockdown or BLU-9931 treatment. β-actin was used as a loading control. **C** Effect of siRNA-mediated FGFR4 knockdown on the gene expression levels of *Ifnα*, *Ifnβ*, and *Cxcl10* induced by FGFR4 overexpression in CT-26 colon cancer cells. **D** Effect of pharmacological inhibition of FGFR4 activity by BLU9931 on the expression levels of *Ifnα*, *Ifnβ*, and *Cxcl10* induced by FGFR4 overexpression in CT-26 colon cancer cells. Data are presented as mean ± SD from three independent experiments. Statistical significance: ns, not significant; **P < 0.01; ***P < 0.001; ****P < 0.0001 using one-way ANOVA. Veh vehicle, BLU BLU9931, siNC siRNA negative control, siF4 siFgfr4.

Western blot analysis confirmed the increased expression of IFN-β and CXCL10 as well as phosphorylation of TBK1, IRFs, and STAT1 in FGFR4-overexpressing colorectal cancer cells (Fig. 4A and Fig. S2a). These findings indicate that the TLR3-TBK-IRF and IFNβ-STAT-CXCL10 signaling pathways were activated by FGFR4 overexpression. Conversely, knockdown of FGFR4 expression using siRNA or treatment with the FGFR4 inhibitor BLU9931 led to a reduction in the phosphorylation levels of TBK1, IRF, and STAT1, as well as a decrease in IFN-β and CXCL10 expression (Fig. 4B and Fig. S2b). Additionally, RT-qPCR analysis demonstrated that FGFR4 knockdown or inhibition significantly reduced the mRNA levels of IFN-β and CXCL10 (Fig. 4C, D and Fig. S2c). The reduction in CXCL10 secretion caused by FGFR4 knockdown was further validated using ELISA analysis (Fig. S2d). These findings collectively indicate that FGFR4 regulates CXCL10 expression and production by activating TLR3-TBK-IRF signaling and the autocrine action of IFNβ in colon cancer cells.

### CXCL10 induces fibroblast differentiation into CAFs

**Fig. 5:**
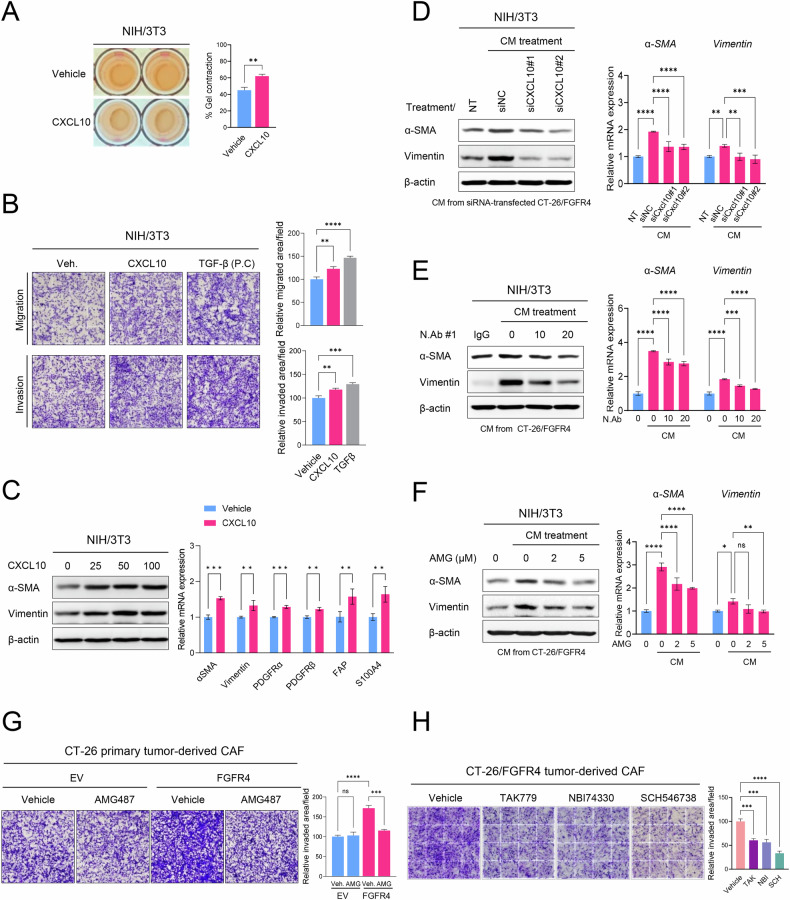
FGFR4 induces CAF differentiation and activation via the CXCL10-CXCR3 axis. **A** Collagen gel contraction assay results show the effect of recombinant CXCL10 protein on the contractility of NIH/3T3 cells (n = 6/group). Representative gel images are shown together with the quantification of collagen gel contraction. **B** Migration and invasion assay results show the effect of recombinant CXCL10 protein on the migration and invasion properties of NIH/3T3 cells. The effect of CXCL10 was compared to that of TGFβ, which induces CAF activation. **C** The effect of recombinant CXCL10 protein (ng) on CAF marker gene levels in NIH/3T3 cells was determined using western blotting and RT-qPCR analyses. **D** Effect of *Cxcl10* knockdown on CAF marker gene expression. NIH/3T3 cells were incubated for 24 h with conditioned media (CM) obtained from siNC- or siCXCL10-transfected CT-26/FGFR4 cells. Western blotting and RT-qPCR analyses of CAF marker expression in NIH/3T3 cells. **E** Effect of CXCL10 neutralizing antibody on CAF marker gene expression. NIH/3T3 cells were incubated in CM obtained from CT-26/FGFR4 cells in the presence of either normal anti-IgG or anti-CXCL10 neutralizing antibodies (concentrations shown in μg/mL) for 24 h. **F** Effect of AMG487, a pharmacological CXCR3 inhibitor, on gene expression of CAF markers. The CM of CT-26/FGFR4 cells, alone or in combination with the CXCR3 inhibitor, was used to treat the NIH/3T3 cells for 24 h. **G** Invasion assay results depicting the effect of the CXCR3 inhibitor AMG487 (2 μM) on the invasion capabilities of CT-26 primary tumor-derived CAFs. **H** Invasion assay results show the effects of various CXCR3 inhibitors on the invasive properties of CT26/FGFR4 tumor-derived CAFs. The CXCR3 inhibitors used (at 2 μM) were: TAK779, NBI74330, and SCH546738. Data are presented as mean ± SD. Statistical significance: *P < 0.05; **P < 0.01; ***P < 0.001; ****P < 0.0001 using Student’s *t*-test and one-way ANOVA). NT no treatment, AMG AMG487, TAK TAK779, NBI NBI74330, SCH SCH54673.

FGFR4-overexpressing cancer cells induced CAF differentiation/activation and exhibited higher concentrations of CXCL10 than control cells (Figs. 2 and 3G). To determine if FGFR4-mediated CXCL10 secretion modulates CAF differentiation and activation in a paracrine manner within the TME, we examined whether CXCL10 directly regulates CAF differentiation/activation in fibroblasts. Treatment with recombinant CXCL10 increased the contractility, migration, invasion, and CAF marker expression in NIH/3T3 fibroblasts, indicating that CXCL10 directly induces fibroblast differentiation into CAFs (Fig. 5A–C). These findings suggest that CXCL10 secreted by FGFR4-overexpressing cells may be critical in regulating CAF differentiation/activation.

### FGFR4 induces CAF differentiation and activation via the CXCL10-CXCR3 axis

We further evaluated whether CXCL10 is critical to secretome-mediated CAF differentiation. α-SMA and vimentin expression was reduced by the CM of si-CXCL10-treated CT-26/FGFR4 cells, compared with that in the si-negative control (NC) group (Fig. 5D, and Fig. S3a). To block secreted CXCL10, we also examined the effects of CXCL10-neutralizing antibodies on CM-mediated CAF differentiation. CXCL10-neutralizing antibodies treatment reduced the CT-26/FGFR4-CM-mediated gene expression of α-SMA and vimentin in NIH/3T3 fibroblasts (Fig. 5E and Fig. S3b). These results suggest that FGFR4 induces CAF differentiation and activation through the secretion of CXCL10.

We examined whether CXCR3, the CXCL10 cognate receptor, contributed to CXCL10-mediated CAF differentiation/activation. Treatment with AMG487, a CXCR3 antagonist, reduced CM-mediated gene expression of α-SMA and vimentin, indicating that the CXCL10-CXCR3 axis plays a central role in CAF differentiation (Fig. 5F). Furthermore, suppression of CXCR3 activity by AMG487 and other CXCR3 inhibitors decreased the invasive capacity of CT-26/FGFR4-derived CAFs (Fig. 5G, H). Collectively, these results demonstrate that FGFR4 overexpression induces CAF differentiation/activation via the CXCL10-CXCR3 axis.

### FGFR4 expression and CAF markers are positively correlated in colon cancer

**Fig. 6:**
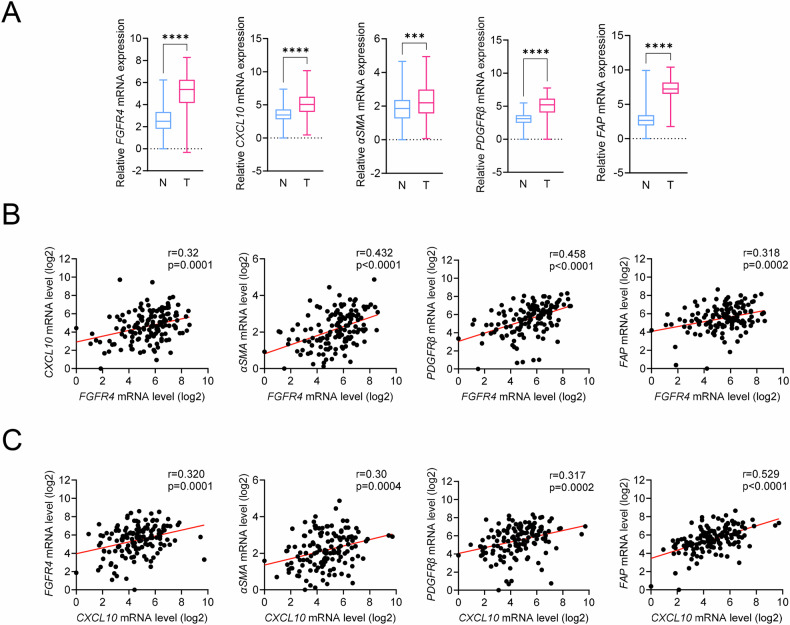
Correlation analysis of CAF markers and FGFR4 or CXCL10 expression in tissue samples from colorectal cancer patients. **A** *FGFR4*, *CXCL10*, and CAF marker mRNA expression in tissue samples from colorectal cancer patients (n = 137) and adjacent normal tissues (n = 137) were analyzed using RT-qPCR. Data presented as the mean ± SD (n = 137 independent samples; statistical analysis via Student’s *t*-test; ***P < 0.001, ****P < 0.0001). **B** Correlation analysis between the expression of *FGFR4* and that of CAF markers, such as *CXCL10*, *αSMA*, *PDGFRβ*, and *FAP* in tissue samples from colorectal cancer patients (n = 137) was performed using Pearson’s correlation test. **C** Correlation analysis between CXCL10 and CAF marker expression in tissue samples from colorectal cancer patients (n = 137), was examined using Pearson’s correlation test. Statistical significance was assessed using Pearson’s correlation coefficient (*r*) and Student’s *t*-test (P-value).

Clinical samples of colon cancer patients were evaluated to assess the correlation between the expression of FGFR4, CXCL10, and CAF markers. Gene expression levels were analyzed in tumors (n = 137) and matched normal tissues (n = 137) from stage II and III colon cancer patients using RT-qPCR. The expression levels of *FGFR4*, *CXCL10*, and CAF markers were significantly upregulated in colon cancer tissues compared with those in normal tissue samples (Fig. 6A). *FGFR4* expression was significantly positively correlated with that of *CXCL10* (Pearson’s R = 0.32; P < 0.001) and CAF markers, including *α-SMA* (Pearson’s R = 0.43; P < 0.0001), *PDGFR*-*β* (Pearson’s R = 0.46; P < 0.0001), and *FAP* (Pearson’s R = 0.32; P < 0.001) in colon cancer samples (Fig. 6B, and Fig. S4a, b). *CXCL10* expression was positively correlated with that of α-SMA (Pearson’s R = 0.30; P < 0.001), *PDGFR-β* (Pearson’s R = 0.32; P < 0.001), and *FAP* (Pearson’s R = 0.53; P < 0.0001) (Fig. 6C, and Fig. S4a, c). These observations strongly support an association between FGFR4, CXCL10, and CAF marker expression in clinical colon cancer samples and show that FGFR4 is involved in CAF differentiation/activation within the colon cancer TME, emphasizing the clinical relevance of the identified FGFR4-CXCL10-CAF regulatory axis.

### Dual inhibition of FGFR4 and CXCR3 suppresses colon cancer growth through CAF inhibition

**Fig. 7:**
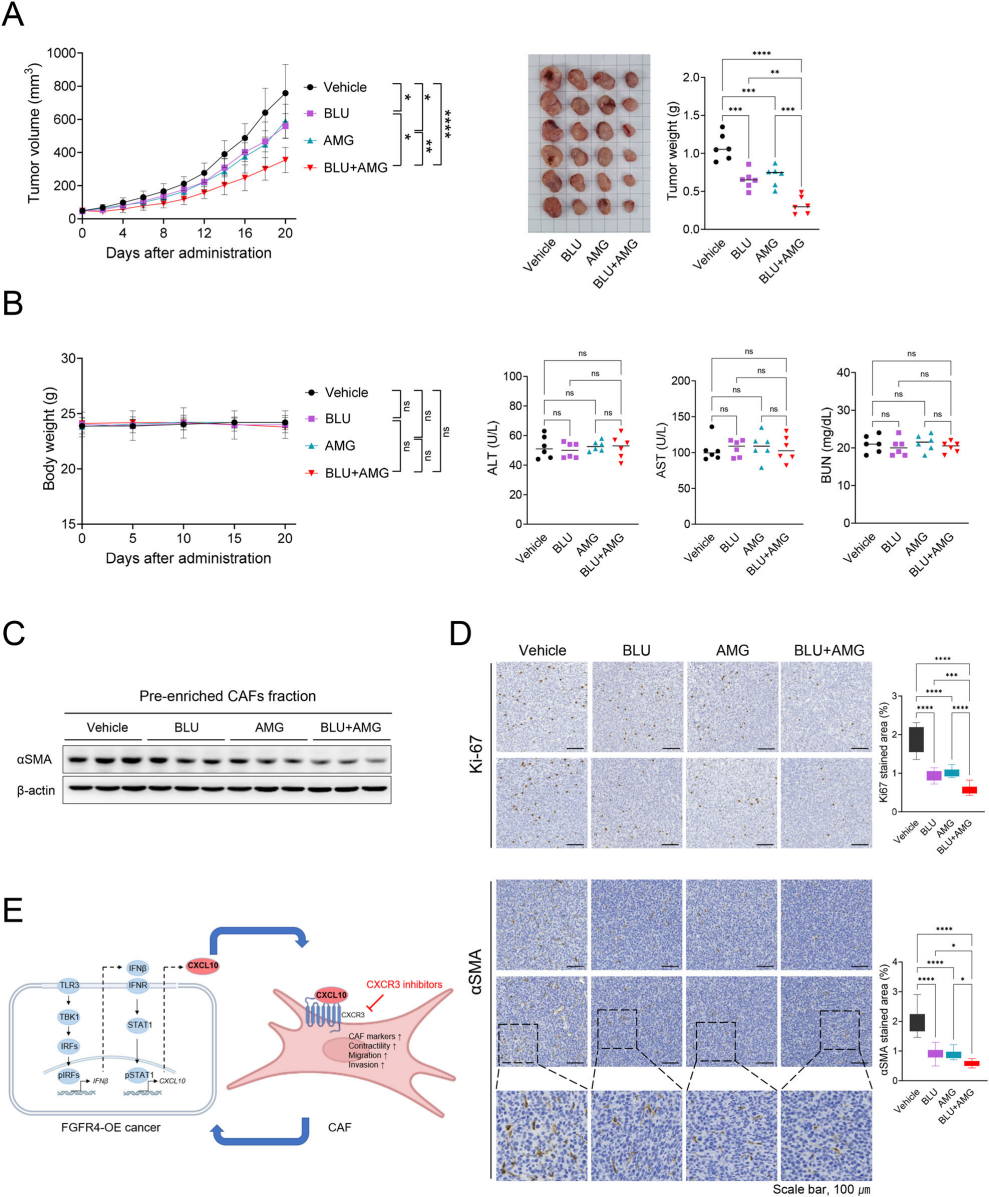
CAF inactivation and antitumor efficiency of FGFR4 and CXCR3 inhibition in vivo. CT-26/FGFR4 cells (2 × 10^5^) were subcutaneously injected into the flanks of Balb/c mice. When tumors reached ~70 mm³, mice were treated with vehicle, BLU9931 (30 mg/kg, oral, twice daily), AMG487 (5 mg/kg, subcutaneously, twice daily), or a combination of both. Tumor samples and blood serum were collected at the end of the study. **A** Left: Average tumor growth curves after the indicated treatment (n = 6/group). Right: Representative tumor image with tumor weight at the end of the study. **B** Left: Body weight changes during the treatment period. Right: Effects of treatments on liver and kidney functions were assessed at the end of the study by measuring serum levels of ALT, AST, and BUN using biochemistry analysis. No statistically significant difference (ns) was observed between groups. **C** Western blotting analysis of α-SMA levels in protein extracts prepared from the tumor-derived pre-enriched CAF fraction. **D** Histopathological analysis of Ki67 and α-SMA of tumors to assess tumor proliferation and CAF distribution. Scale bar: 100 μm. **E** Schematic of the working model for FGFR4-mediated induction of CAF differentiation/activation in colorectal cancer. Overexpressed FGFR4 induces CXCL10 secretion via activation of the TLR3-TBK-IRF signaling pathway and autocrine action of IFNβ in colon cancer cells. Subsequently, FGFR4 overexpression facilitates the differentiation/activation of CAFs via the CXCL10-CXCR3 axis. Data are presented as the mean ± SD. Statistical significance: *P < 0.05; **P < 0.01; ***P < 0.001; ****P < 0.0001 using one-way ANOVA.

We investigated whether treatment with FGFR4 and CXCR3 inhibitors can suppress tumor growth and CAF differentiation. Treatment with BLU9931, an FGFR4 inhibitor, attenuated tumor progression, and co-treatment with the CXCR3 inhibitor AMG487 further enhanced this inhibitory effect, highlighting the synergistic function of the two inhibitors (Fig. 7A). We also assessed the potential toxicity of the dual treatment by monitoring body weight and performing blood biochemistry analysis. No significant toxicity was observed in treatment groups, as indicated by consistent body weights and normal blood biochemistry parameters, including ALT, AST, and BUN (Fig. 7B). These results suggest that the dual treatment as well as single treatment did not induce systemic toxicity. Western blot analysis of tumor-derived pre-enriched CAF fractions (stromal enrichment fraction) showed the decreased expression of α-SMA following dual treatment with BLU9931 and AMG487, indicating CAF suppression (Fig. 7C). IHC analysis supported the dual inhibition of FGFR4 and CXCR3, which reduced the number of α-SMA- and Ki-67-positive cells within the TME, suggesting attenuation of CAF differentiation and tumor proliferation (Fig. 7D).

In addition to CAF suppression, we assessed the immune-modulating effects of dual FGFR4 and CXCR3 inhibition through IHC staining for CD8, CD86, and CD206. Dual treatment increased CD8^+^ T cell infiltration and reduced CD206^+^ tumor-associated macrophage (TAM), indicating a more immune-activating environment (Fig. S5). Moreover, levels of CD86^+^ cells, indicating M1 macrophages, were elevated in the combination therapy group. These findings suggest that dual treatment, by suppressing CAF differentiation, may enhance CD8^+^ T cell infiltration and shift the immune response towards a more anti-tumor phenotype. Collectively, dual inhibition of FGFR4 and CXCR3 suppressed tumor growth in vivo, which was accompanied by CAF suppression and immune modulation in TME.

## Discussion

The cytokine-chemokine network and its receptor can bi-directionally modulate TME, potentially promoting tumor progression, metastasis, and therapy resistance through the enhanced attraction of immune cells and the induction of cell differentiation or vice versa. In fact, the same cytokines or chemokines can have either antitumor or protumor activities depending on their effector functions and immune context. The CXCL-9, −10, −11/CXCR3 axis also exhibits bifunctional activities within TME. These chemokines are primarily secreted by monocytes, endothelial cells, fibroblasts, and tumor cells in response to IFN-γ, with their secretion being synergistically enhanced by tumor necrosis factor alpha (TNFα) [17, 18]. CXCR3, the cognate receptor for CXCL9, −10, −11, is predominantly expressed on monocytes as well as T, natural killer (NK), dendritic, and tumor cells [23, 24]. The CXCL-9, −10, −11/CXCR3 axis promotes tumor suppression through the recruitment of cytotoxic lymphocytes, NK cells, and NK T cells in a paracrine manner. Furthermore, this axis induces Th1 polarization and activates immune cells in response to IFN-γ [25]. Their expression is positively correlated with the antitumor activity of CD8^+^ T cells. However, recent studies revealed that the autocrine CXCL9, −10, −11/CXCR3 axis in tumor cells facilitates proliferation, angiogenesis, and metastasis as well as reduced CXCR3 expression on CD8^+^ T cells [26–29]. Furthermore, the CXCL-10/CXCR3 axis was shown to enhance tumorigenicity and EMT induction in colon cancer cells [30]. Fibroblast-derived CXCL9/10 also promotes lung metastasis by stimulating the growth of CXCR3^+^ cancer cells. Blocking the CXCL9/10-CXCR3 axis through CXCR3 inhibition has previously been proposed as a strategy for suppressing tumor metastasis to the lungs in breast cancer [31]. Thus, the effects of the CXCL9, −10, −11/CXCR3 axis depend on the target effector cells and should be evaluated in comprehensive and dynamic evaluation. Our current data demonstrate that FGFR4 induces CXCL10 production in colon cancer cells and that the secreted CXCL10 promotes CAF differentiation in a paracrine manner. CXCL10 neutralizing antibodies or CXCR3 inhibitors eliminated the expression of CAF markers and attenuated CAF function. These findings highlight the pro-tumoral function of CXCL10/CXCR3 signaling in colon cancer through the induction of CAF differentiation and activation.

CAFs are fibroblast populations located within primary and metastatic cancer tissues. CAF activation is determined by ECM synthesis and remodeling, secretory capacity, proliferation, and the expression of markers such as α-SMA and vimentin. CAFs serve as a physical and chemical barrier, protecting cancer cells from immune surveillance and secreting immunosuppressive cytokines [32]. CAFs shape the ECM architecture, which acts as a physical barrier to anticancer drug delivery and CD8^+^ T-cell infiltration [33]. CAF-derived cytokines, including IL-6, GM-CSF, and CXCL12, induced the differentiation and activation of myeloid-derived suppressor cells (MDSCs) and TAMs to favor cancer progression [34, 35]. CAFs also recruited and stimulated DCs to acquire a tolerogenic phenotype through IL-6 secretion [36]. Considering their tumor-promoting functions, numerous CAF-targeting strategies are being evaluated in pre-clinical and clinical studies [37, 38]. However, several of these, such as targeting hyaluronic acid, did not exhibit sufficient therapeutic efficacy [39, 40]. Therefore, the underlying mechanism and regulator of CAF differentiation and activation should be studied in-depth to allow a better-defined set of biomarkers and further therapeutic approaches targeting CAFs. Using bioinformatics analysis, Lu et al. suggested that the CAF score can predict immunotherapy response and identified FGFR4 as a CAF-related protein in pancreatic cancer [41]. To the best of our knowledge, this is the first report to reveal the interplay between FGFR4 in colon cancer cells and the CXCL10/CXCR3 axis in CAFs, demonstrating the efficacy of dual FGFR4 and CXCR3 inhibition against tumor growth via CAF suppression. Since CXCL10 exerts immunostimulatory effects on cytotoxic lymphocytes within TME, we used the CXCR3 inhibitor instead of an anti-CXCL10 blocking antibody, which may attenuate the antitumor effect. To achieve a maximal therapeutic response in combination with an FGFR4 inhibitor, further research is needed to investigate CXCR3 inhibitors, focusing on the optimal inhibitory compound, concentration, and CAF-specific delivery. This approach can avoid undesired bystander effects of CXCL10-CXCR3 in immune TME and could be extended to other types of solid tumors for controlling both CAFs and tumor cells.

In conclusion, FGFR4 in cancer cells promotes CXCL10 production via TLR3-IRF-IFNβ-dependent signaling that subsequently induces CAF differentiation and activation, culminating in colon cancer progression. Dual inhibition of FGFR4 and CXCR3 suppresses tumor growth through CAF downregulation. Thus, CAF suppression via FGFR4/CXCR3 inhibition holds the potential for overcoming treatment resistance.

## Supplementary information

Supplementary information

Supplementary figure 1

Supplementary figure 2

Supplementary figure 3

Supplementary figure 4

Supplementary figure 5

Original WB figure-1

Original WB figure-2

Original WB figure-3

Original WB figure-4

Original WB figure-5

Original WB figure-6

## Data Availability

The data supporting the findings of this study are available from the corresponding author upon reasonable request.
