# Supplementary material for: FGFR4 promotes CAF activation through the CXCL10-CXCR3 axis in colon cancer
Source: Cell Death Dis. 2025 May 30;16(1):424. doi: 10.1038/s41419-025-07588-y (PMC12125224; doi:10.1038/s41419-025-07588-y)
Supplement: Supplementary file 1 — Supplementary information [file 41419_2025_7588_MOESM1_ESM.docx]

**Supplementary Information**

**Supplementary Tables**

**Table S1: Primers used for plasmid construction.**

| Primer name | Sequence (5′-3′) | Vector |
| --- | --- | --- |
| mFgfr4-F | GCTGGCTAGTTAAGCTTGGATGTGGCTGCTCTTGGCCCTG | pcDNA6/myc-His A |
| mFgfr4-R | GGCCCTCTAGACTCGAGTGTCGTCTGCGAGTCAGAGAAAG |  |
| hFGFR4-F | CCCAAGCTTGGAATGCGGCTGCTGCTGGCCCTGTTGG | pcDNA6/myc-His A |
| hFGFR4-R | CCGCTCGAGTGTCTGCACCCCAGACCCGAAGGGGA |  |

**Table S2: Primers used for RT-qPCR.**

| **Mouse Gene** | **Primer sequence** | |
| --- | --- | --- |
|  | **Forword (5′-3′)** | **Reverse (3′-5′)** |
| Tlr3  (NM_126166) | GTCTTCTGCACGAACCTGACAG | TGGAGGTTCTCCAGTTGGACCC |
| Ifn-α  (NM_010502) | GAGAAGAAACACAGCCCCTG | TCAGTCTTCCCAGCACATTG |
| Ifn-β  (NM_010510) | CAGCTCCAAGAAAGGACGAAC | GGCAGTGTAACTCTTCTGCAT |
| Cxcl10  (NM_021274) | TTTCTGCCTCATCCTGCTG | CGCAGGGATGATTTCAAG |
| α-Sma  (NM_007392) | TGCTGACAGAGGCACCACTGAA | CAGTTGTACGTCCAGAGGCATAG |
| Pdgfrα  (NM_001083316) | GCAGTTGCCTTACGACTCCAGA | GGTTTGAGCATCTTCACAGCCAC |
| Pdgfrβ  (NM_008809) | GTGGTCCTTACCGTCATCTCTC | GTGGAGTCGTAAGGCAACTGCA |
| Fap  (NM_007986) | CACCTGATCGGCAATTTGTG | CCCATTCTGAAGGTCGTAGATGT |
| Vimentin  (NM_011701) | CGGAAAGTGGAATCCTTGCAGG | AGCAGTGAGGTCAGGCTTGGAA |
| Fgfr4  (NM_008011) | CAAGTGGTTCGTGCAGAGG | CTTCATCACCTCCATCTCGG |
| Gapdh  (NM_008084) | AGGTCGGTGTGAACGGATTTG | TGTAGACCATGTAGTTGAGGTCA |
| **Human Gene** | **Primer sequence** | |
|  | **Forword (5′-3′)** | **Reverse (3′-5′)** |
| CXCL10  (NM_001565) | GGTGAGAAGAGATGTCTGAATCC | GTCCATCCTTGGAAGCACTGCA |
| α-SMA  (NM_001141945) | CTATGCCTCTGGACGCACAACT | CAGATCCAGACGCATGATGGCA |
| PDGFRα  (NM_006206) | GACTTTCGCCAAAGTGGAGGAG | AGCCACCGTGAGTTCAGAACGC |
| PDGFRβ  (NM_002609) | GAGACTGTTGGGCGAAGGTTA | GAGATGGTTGAGGAGGTGTTGAC |
| FAP  (NM_004460) | GGAAGTGCCTGTTCCAGCAATG | TGTCTGCCAGTCTTCCCTGAAG |
| Vimentin  (NM_003380) | GACGCCATCAACACCGAGTT | CTTTGTCGTTGGTTAGCTGGT |
| FGFR4  (NM_002011) | GCTGGCTTAAGGATGGACAG | CGTTGATGACGATGTGCTTC |
| 18s rRNA  (NR_003286) | CGGCGACGACCCATTCGAAC | GAATCGAACCCTGATTCCCCGTC |
| GAPDH  (NM_002046) | GTGAAGGTCGGAGTCAAC | GTTGAGGTCAATGAAGGG |

**Table S3: Primary antibodies used in the study.**

| **Antibody** | **Manufacturer** | **Cat. No.** | **Application** | **Dilution** |
| --- | --- | --- | --- | --- |
| FGFR4 | CST | 8562 | WB | 1:1000 |
| pFRS2 | CST | 3861 | WB | 1:1000 |
| FRS2 | Thermo Fisher | PA5-27875 | WB | 1:1000 |
| α-SMA | CST | 19245 | WB, IHC/ IF | 1:1000/ 1:200 |
| α-SMA | Abcam | Ab5694 | WB | 1:1000 |
| FAP | Invitrogen | PA5-99313 | WB | 1:1000 |
| PDGFRα | CST | 3174 | WB | 1:1000 |
| Vimentin | CST | 5741 | WB | 1:1000 |
| TLR3 | Abcam | Ab13915 | WB | 1:1000 |
| pTBK1 | CST | 5483 | WB | 1:1000 |
| TBK1 | CST | 3013 | WB | 1:1000 |
| pIRF3 | CST | 29047 | WB | 1:1000 |
| IRF3 | CST | 4302 | WB | 1:1000 |
| pIRF7 | CST | 14767 | WB | 1:1000 |
| pIRF7 | CST | 12390 | WB | 1:1000 |
| IRF7 | CST | 39656 | WB | 1:1000 |
| IRF7 | CST | 13014 | WB | 1:1000 |
| IFN-β (h) | CST | 73671 | WB | 1:1000 |
| IFN-β (M) | CST | 97450 | WB | 1:1000 |
| pSTAT1 | CST | 9167 | WB | 1:1000 |
| STAT1 | CST | 9172 | WB | 1:1000 |
| CXCL10 | Mybiosource | MBS8502352 | WB | 1:500 |
| β-actin | Abcam | ab6276 | WB | 1:1000 |
| β-actin | Abcam | ab8227 | WB | 1:1000 |
| Ki-67 | Abcam | Ab16667 | IHC | 1:2000 |
| CXCL10 | R&D | MAB466 | Neutralization |  |
| CXCL10 | R&D | AF466 | Neutralization |  |
| Alexa488 Phalloidin | Invitrogen | A12379 | IF | 1:40 |
| Goat anti-Rabbit IgG Alexa594 | Invitrogen | A11012 | IF | 1:200 |

All secondary HRP-conjugated antibodies for western blotting analysis were purchased from Jackson ImmunoResearch Laboratories. Abbreviations: h, human; m, mouse; WB, Western blot; IHC, Immunohistochemistry; IF, Immunofluorescence; CST, Cell Signaling Technology.

**Supplementary Figures**

**Fig. S1. Confirmation of FGFR4 overexpression in stable cells.** **Extended data related to Figure 3d.** An established stable cell was used for this study and RNA sequencing. FGFR4 overexpression was determined at the mRNA levels by RT-qPCR in CT-26 and HT-29 cells. Data are presented as mean ± SD. ****p < 0.0001.

**Fig. S2. The role of FGFR4 in the TLR3-TBK-IRF and IFN signaling pathway in HCT116 cells.** (a) Western blot analysis showing the activation of the TLR3-TBK-IRF axis and IFN signaling pathway in HCT116 cells with FGFR4 overexpression (EV: empty vector, FGFR4: overexpression). FGFR4 overexpression led to increased phosphorylation of TBK1, IRF3, IRF7 and STAT1, as well as elevated IFNβ and CXCL10. β-actin was used as a loading control. (b) Western blot analysis showing the effects of FGFR4 knockdown (siNC: negative control siRNA, siF4: FGFR4 siRNA) and FGFR4 inhibition (BLU9931, 1 μM) in FGFR4-overexpressing HCT116 cells. Knockdown of FGFR4 and treatment with BLU9931 reduced the phosphorylation levels of TBK1, IRF3, IRF7, and STAT1, as well as IFNβ and CXCL10 expression. β-actin was used as a loading control. **(c)** Supplementary data related to Figure 4c. Relative expression of FGFR4 mRNA in CT-26 cells following siRNA transfection (siNC: non-targeting control, siF4: FGFR4 siRNA) was assessed by RT-qPCR to confirm the siRNA-mediated FGFR4 knockdown. FGFR4 expression was significantly reduced in siRNA-transfected cells. Data are presented as mean ± SD. ****p < 0.0001. **(d)** CXCL10 production in the conditioned media (CM) of CT-26/FGFR4 stable cells was determined by ELISA. FGFR4 knockdown (siF4) and siCXCL10 transfection both significantly reduced CXCL10 levels compared to the control. Data are presented as mean ± SD. **p < 0.01, ****p < 0.0001.

**Fig. S3. Extended data related to Figure 5.** (a) Supplementary data related to Figure 5d. Cxcl10 knockdown in CT-26/FGFR4 cells was confirmed using RT-qPCR. CXCL10 production was further assessed using ELISA in the conditioned media (CM) of siRNA-transfected stable cells. (b) Supplementary data related to Figure 5e. The effect of CXCL10 neutralizing antibody (Ab) on α-SMA and vimentin gene expression in NIH/3T3 fibroblast. NIH/3T3 cells were incubated with a CM of CT-26/FGFR4 with normal IgG or CXCL10 neutralizing Ab (R&D, AF466) for 24 h. The gene expression was determined by western blotting and RT-qPCR.

**Fig. S4. Extended data related to Figure 6.** (a) Table showing Pearson correlation coefficients and *p* values (r; p) between the gene expression of *FGFR4* and the target genes for all the samples analyzed. (b) Correlation analysis between *FGFR4* and *vimentin* or *PDGFRα* in colorectal cancer (n=137) (c) Correlation analysis between *CXCL10* and *vimentin* or *PDGFRα* in colorectal cancer (n=137).

**Fig. S5. Immunohistochemistry analysis of tumor tissues for immune cell markers following FGFR4 and CXCR3 inhibition in CT-26/FGFR4-tumor bearing mice.** IHC staining for CD8, CD86, and CD206 in tumor tissues from the following treatment groups: control (vehicle), FGFR4 inhibitor (BLU9931), CXCR3 inhibitor (AMG9931), and combination of FGFR4 and CXCR3 inhibitors. CD8, CD86, and CD206 staining indicated the presence of cytotoxic T cells, M1 macrophages, and M2 macrophages within the tumor, respectively. Quantification of the IHC staining is shown as box plots beside the representative images. Data are presented as mean ± SD, and statistical significance is indicated as follows: ns: no significant; *p < 0.05; **p < 0.01; *****p < 0.001;** ******p < 0.001.**
