# Supplementary figures and images for: FGFR4 promotes CAF activation through the CXCL10-CXCR3 axis in colon cancer
Source: Cell Death Dis. 2025 May 30;16(1):424. doi: 10.1038/s41419-025-07588-y (PMC12125224; doi:10.1038/s41419-025-07588-y)

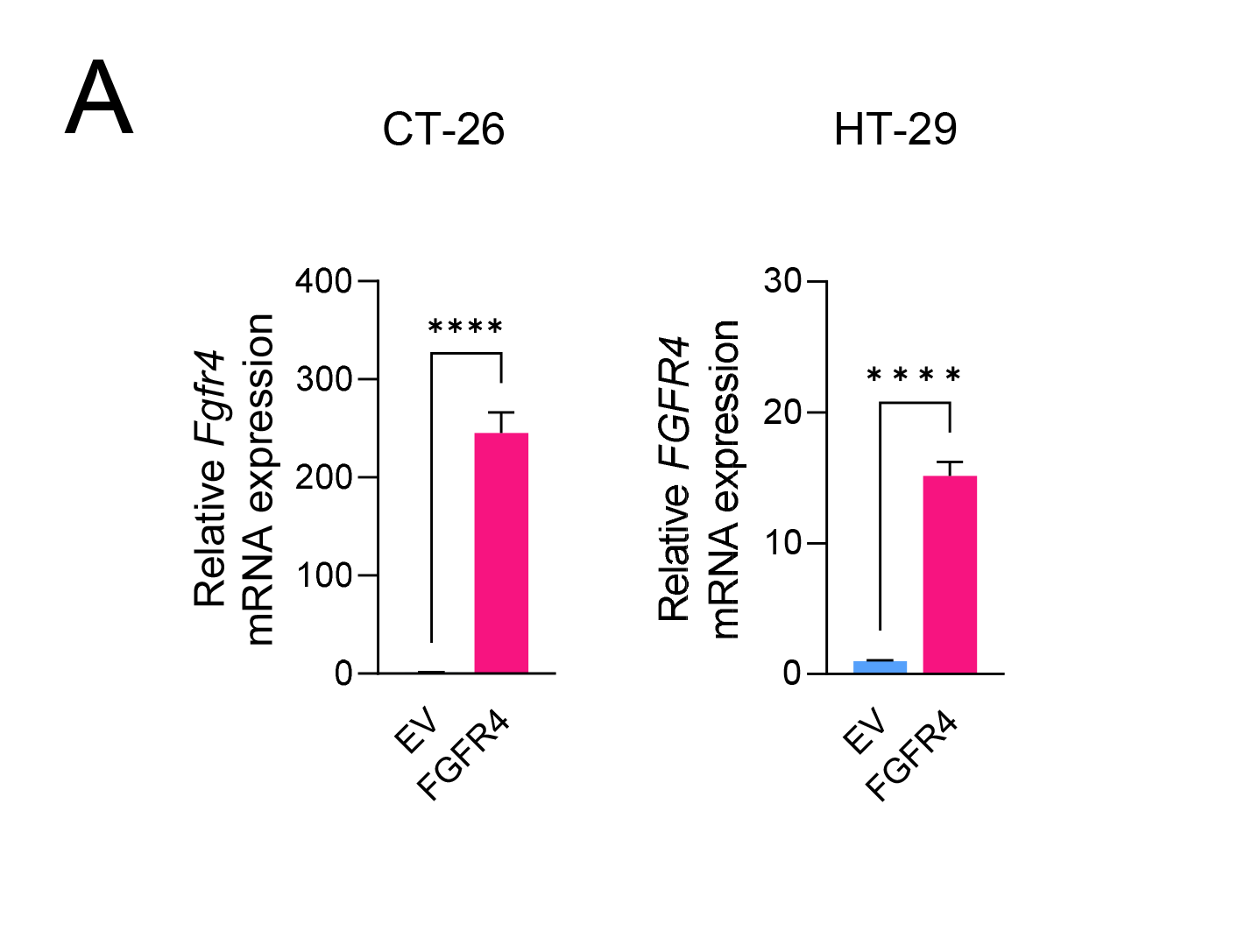

Supplement: Supplementary file 2 — Supplementary figure 1 [file 41419_2025_7588_MOESM2_ESM.tif]

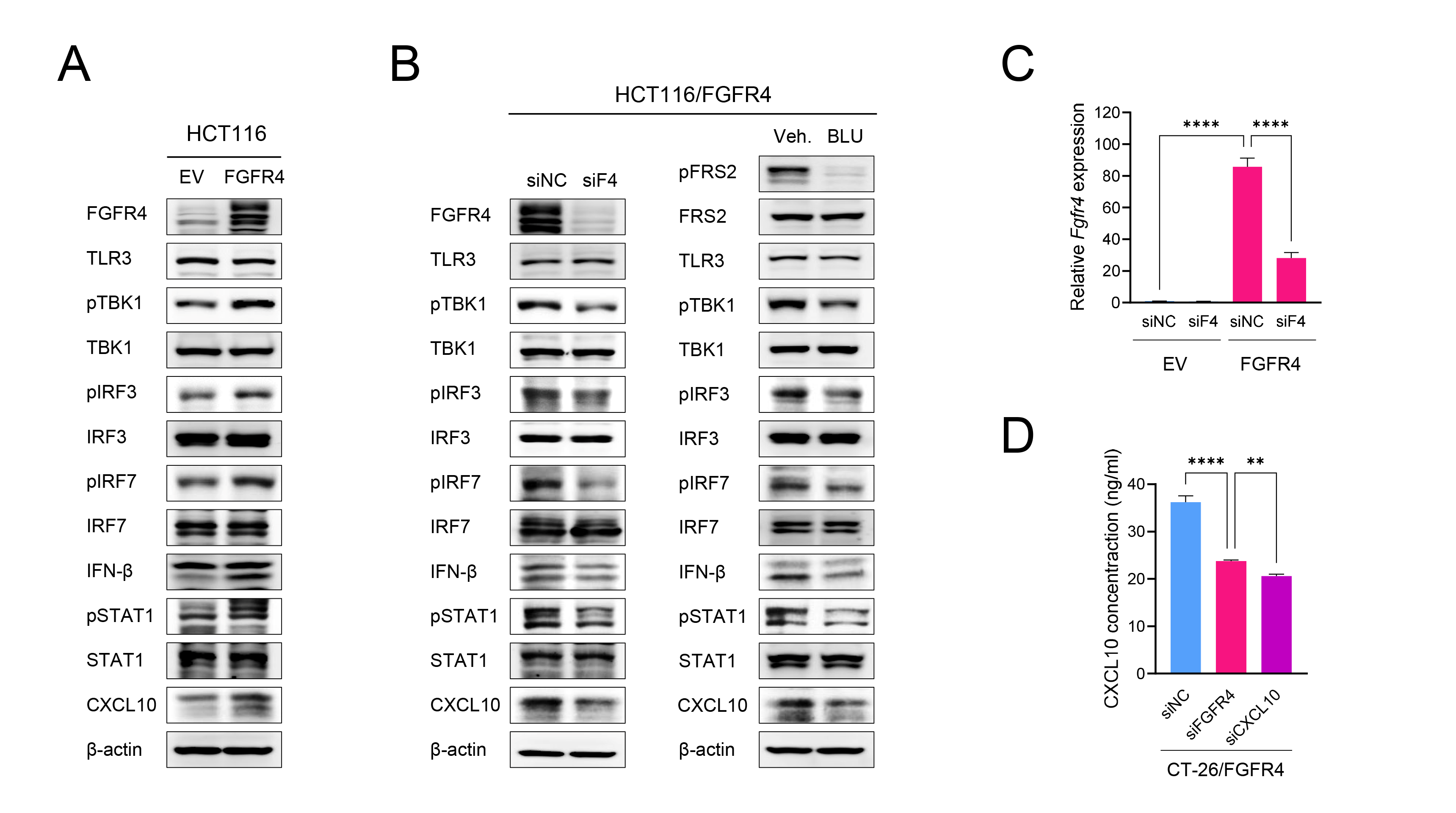

Supplement: Supplementary file 3 — Supplementary figure 2 [file 41419_2025_7588_MOESM3_ESM.tif]

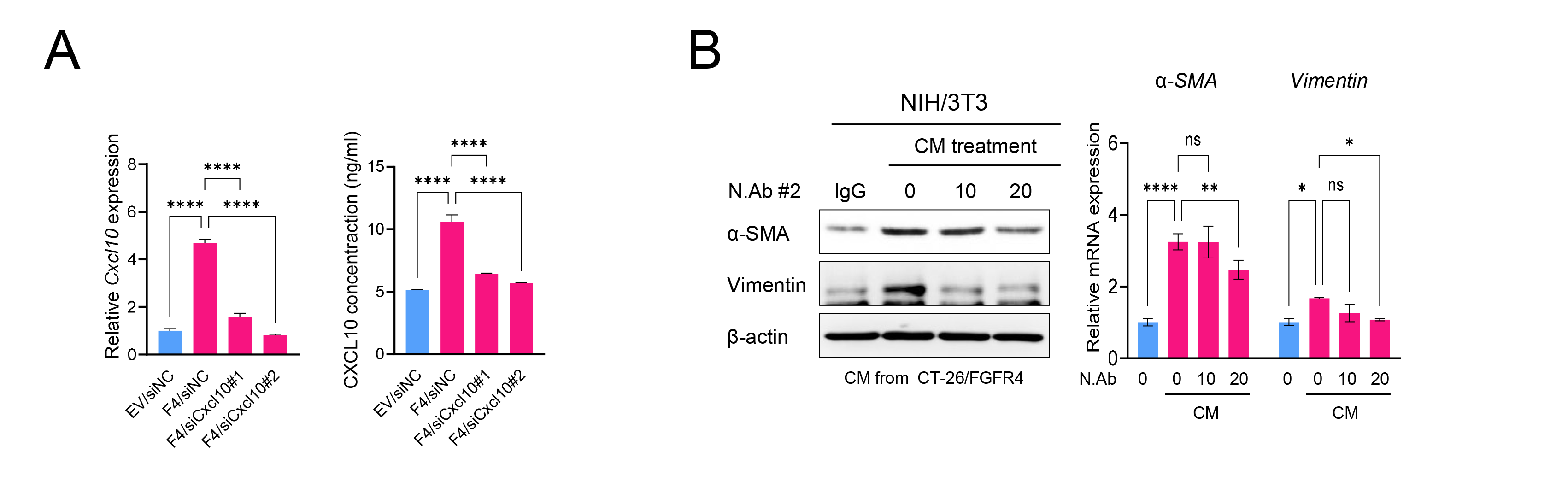

Supplement: Supplementary file 4 — Supplementary figure 3 [file 41419_2025_7588_MOESM4_ESM.tif]

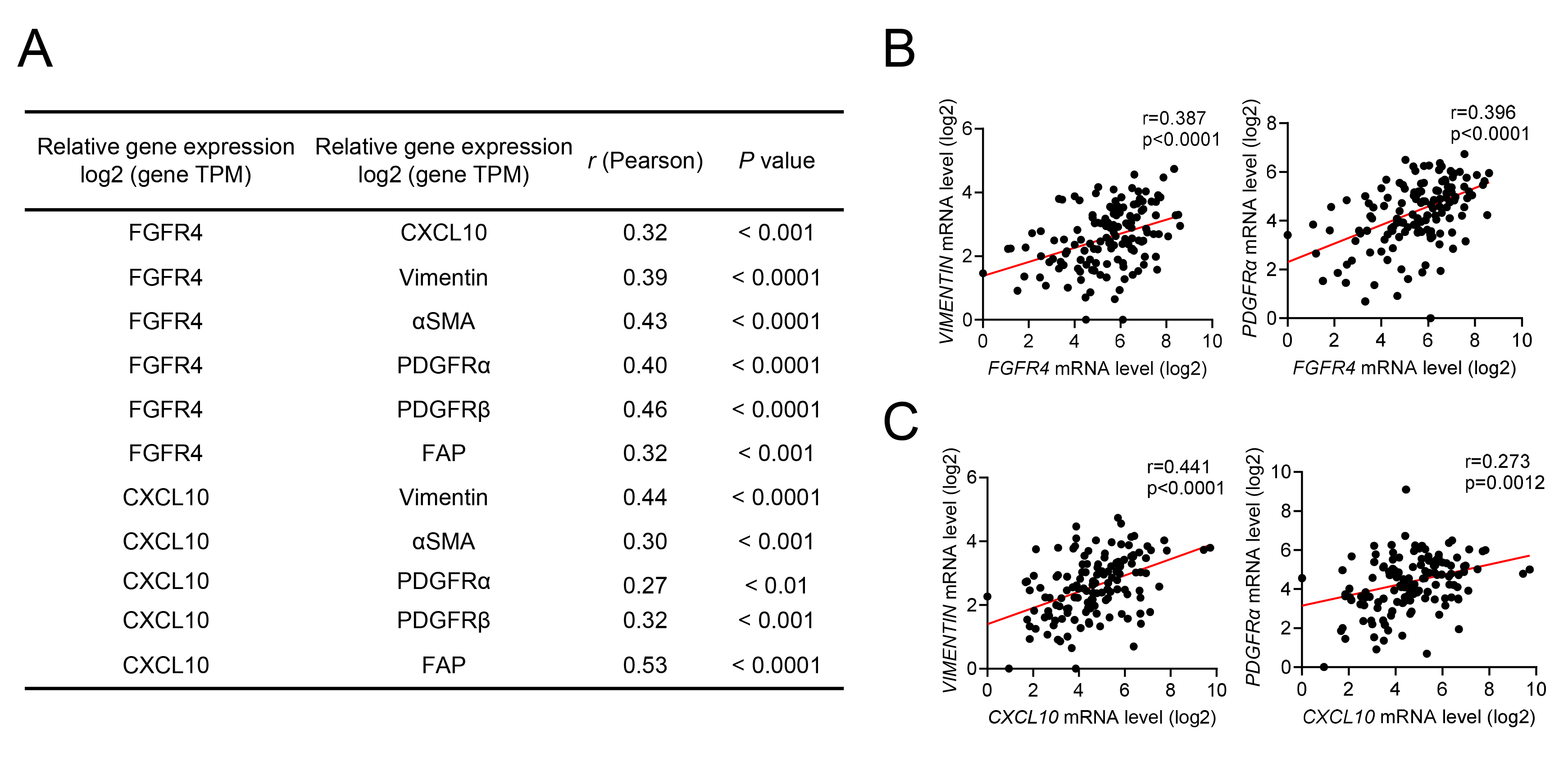

Supplement: Supplementary file 5 — Supplementary figure 4 [file 41419_2025_7588_MOESM5_ESM.tif]

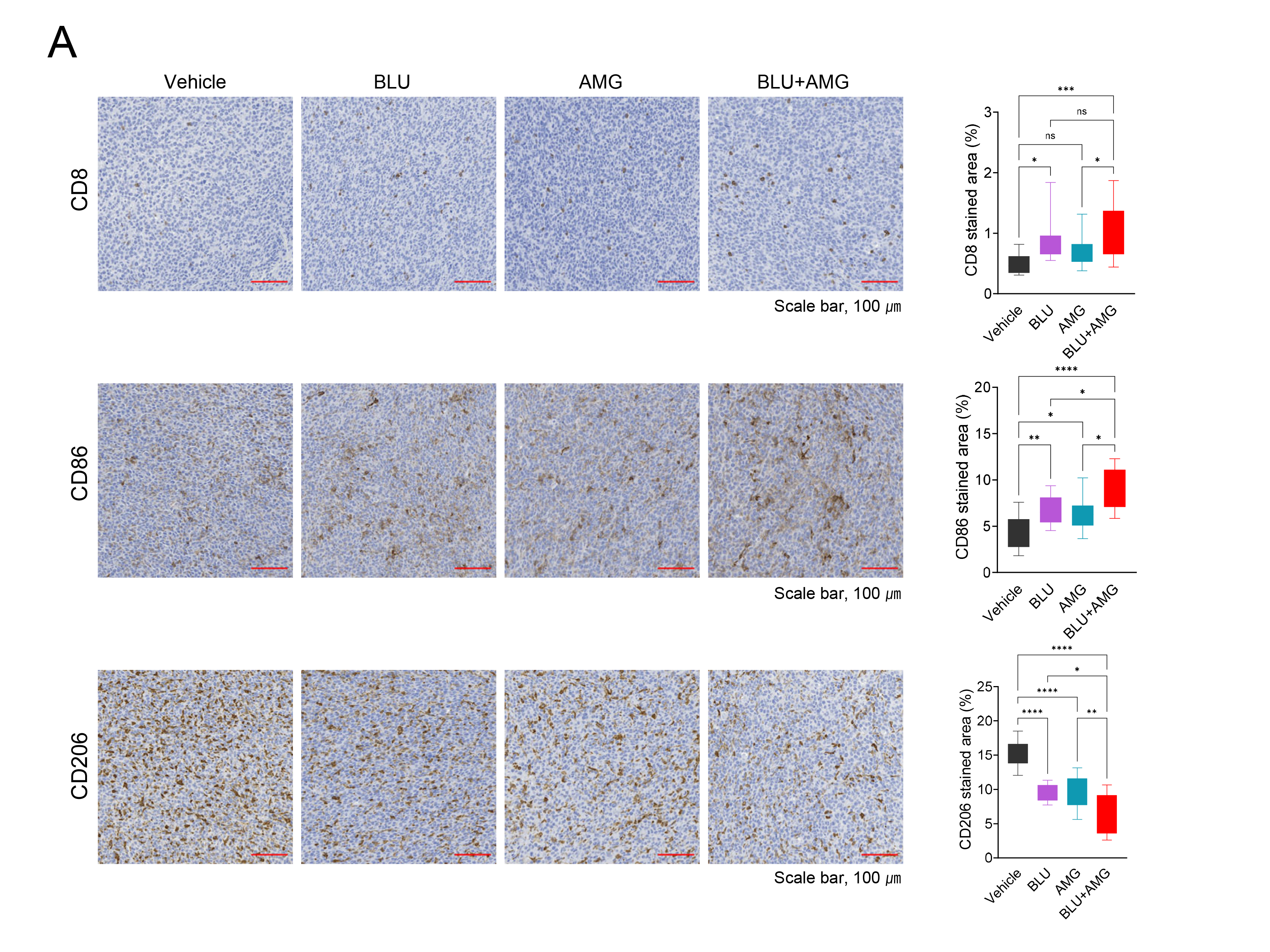

Supplement: Supplementary file 6 — Supplementary figure 5 [file 41419_2025_7588_MOESM6_ESM.tif]

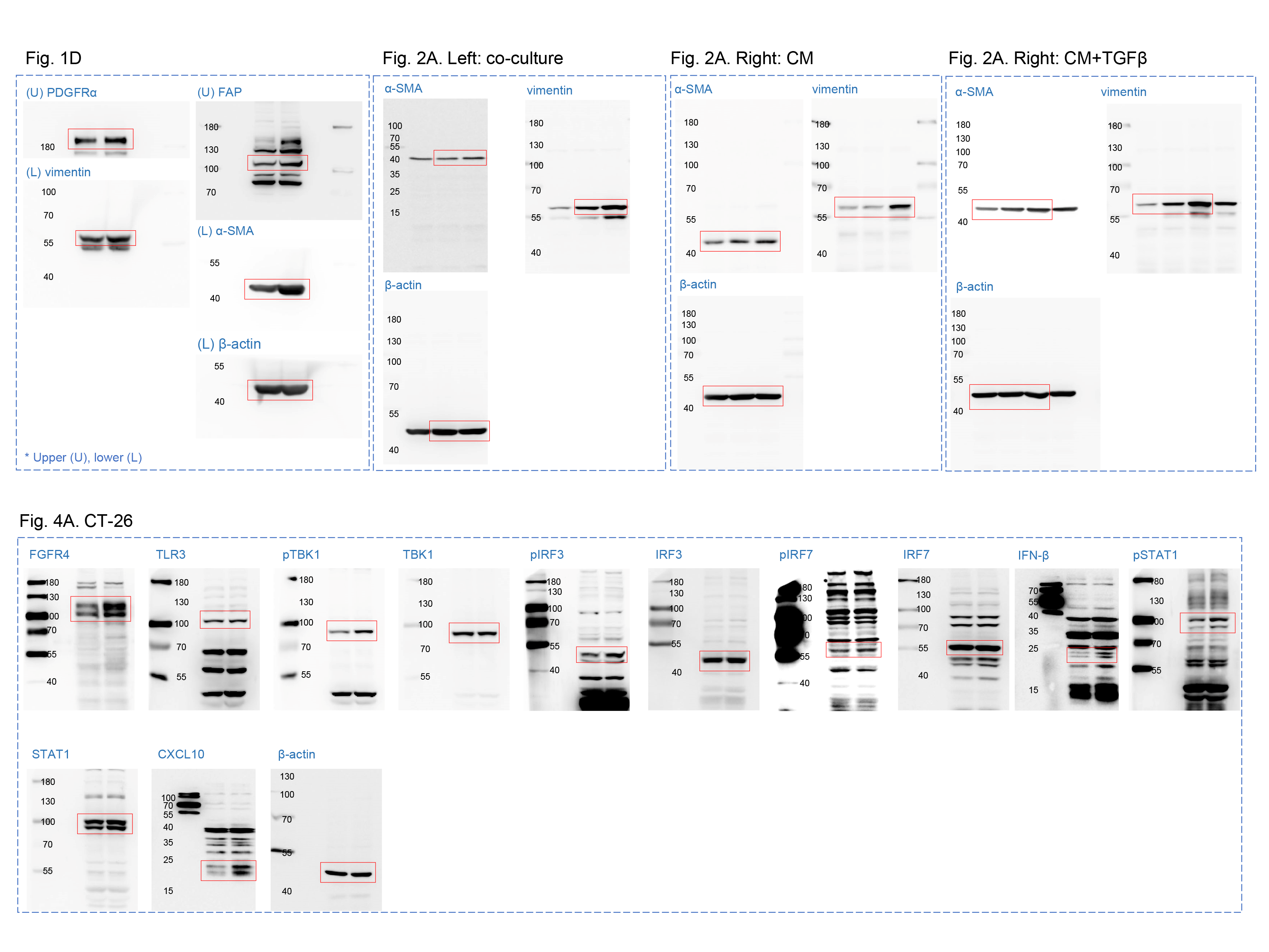

Supplement: Supplementary file 7 — Original WB figure-1 [file 41419_2025_7588_MOESM7_ESM.tif]

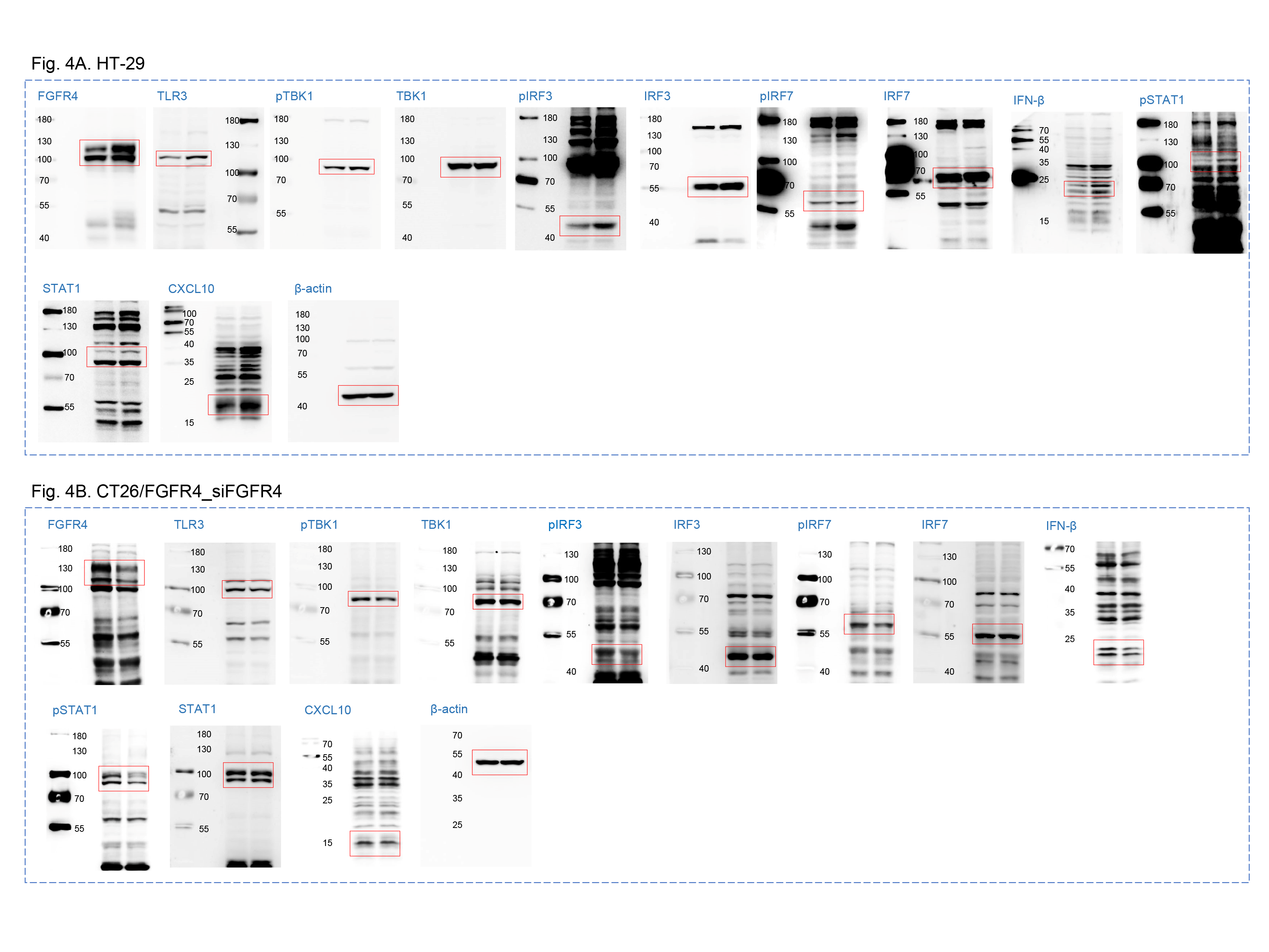

Supplement: Supplementary file 8 — Original WB figure-2 [file 41419_2025_7588_MOESM8_ESM.tif]

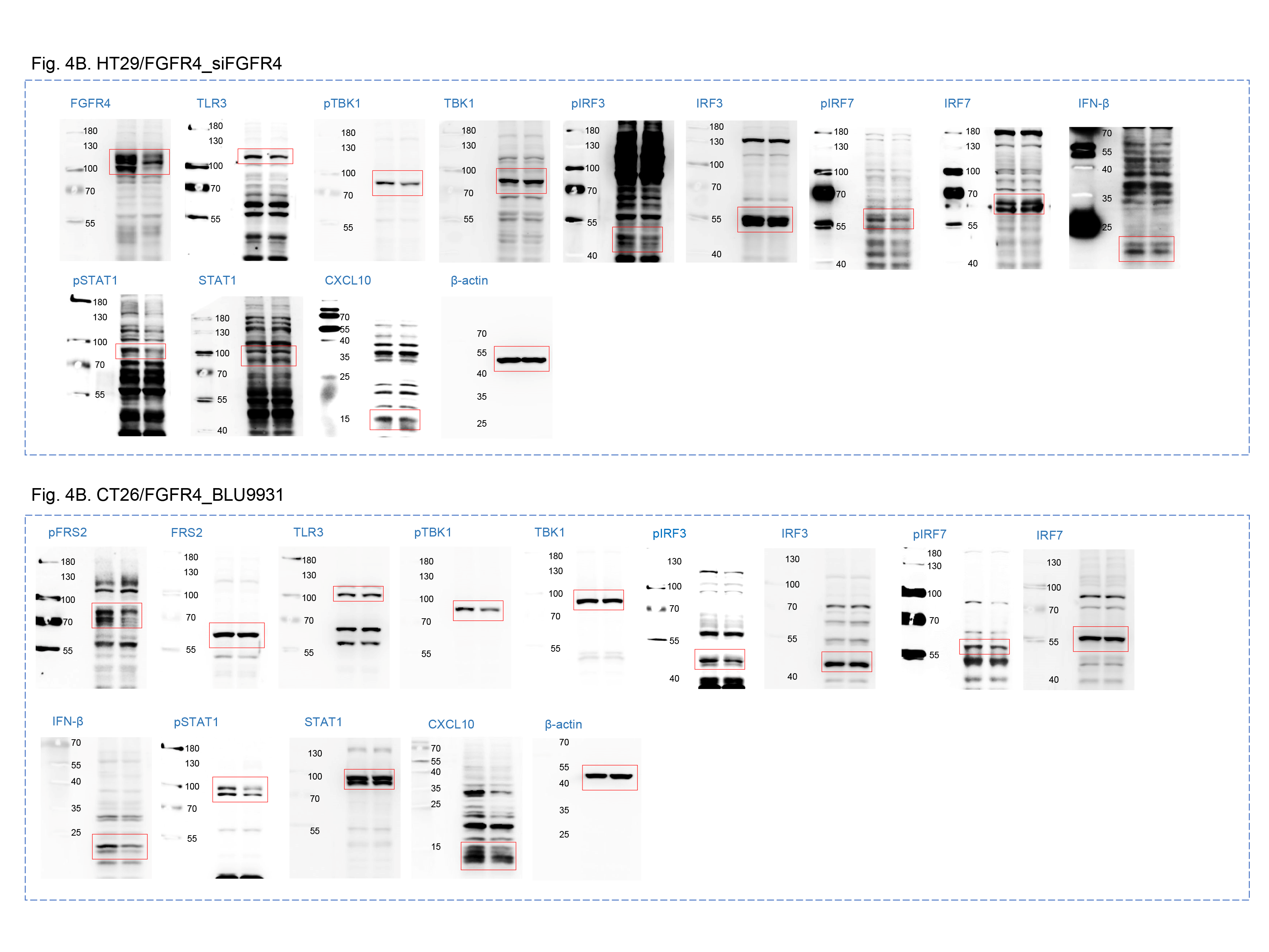

Supplement: Supplementary file 9 — Original WB figure-3 [file 41419_2025_7588_MOESM9_ESM.tif]

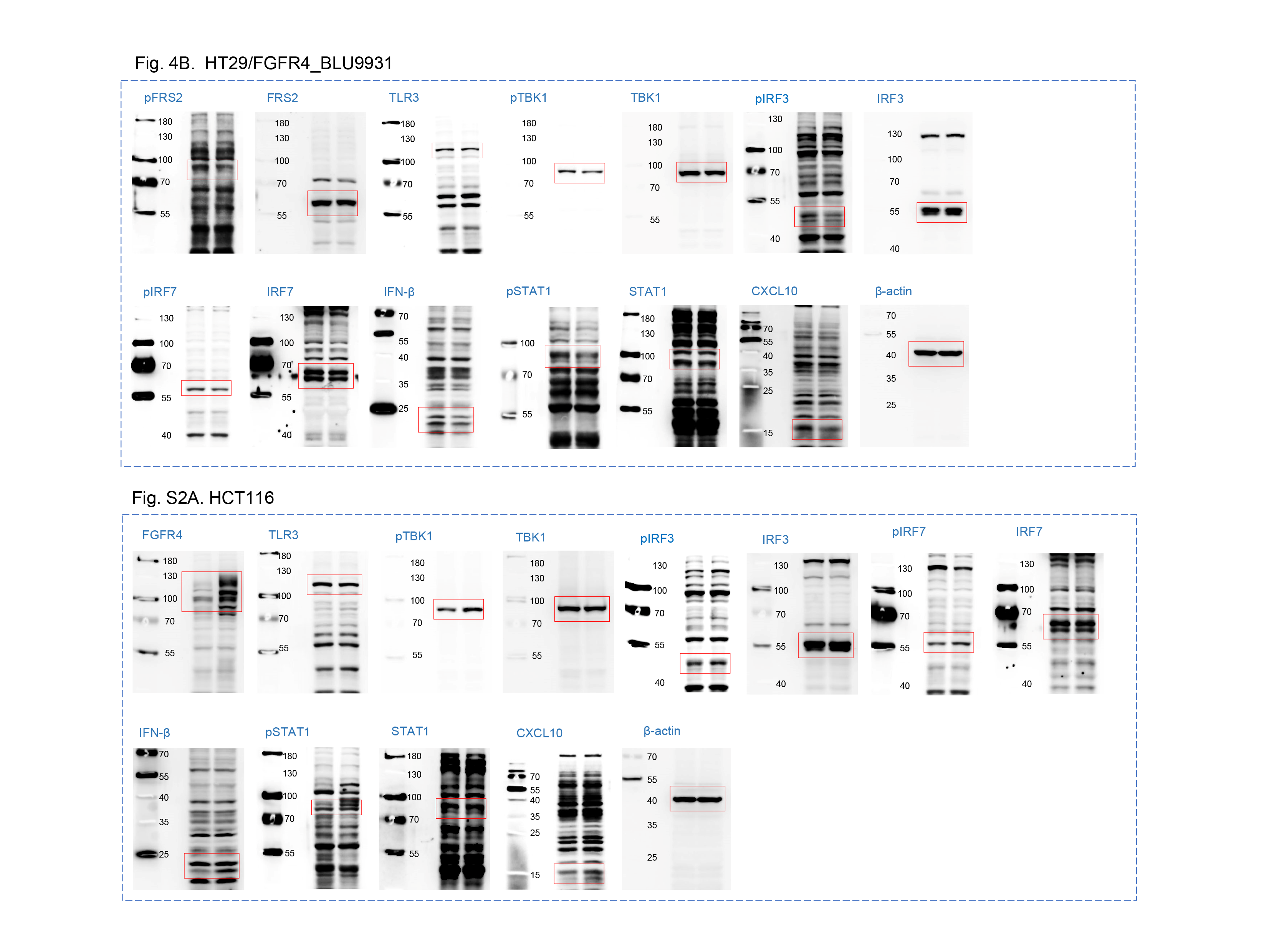

Supplement: Supplementary file 10 — Original WB figure-4 [file 41419_2025_7588_MOESM10_ESM.tif]

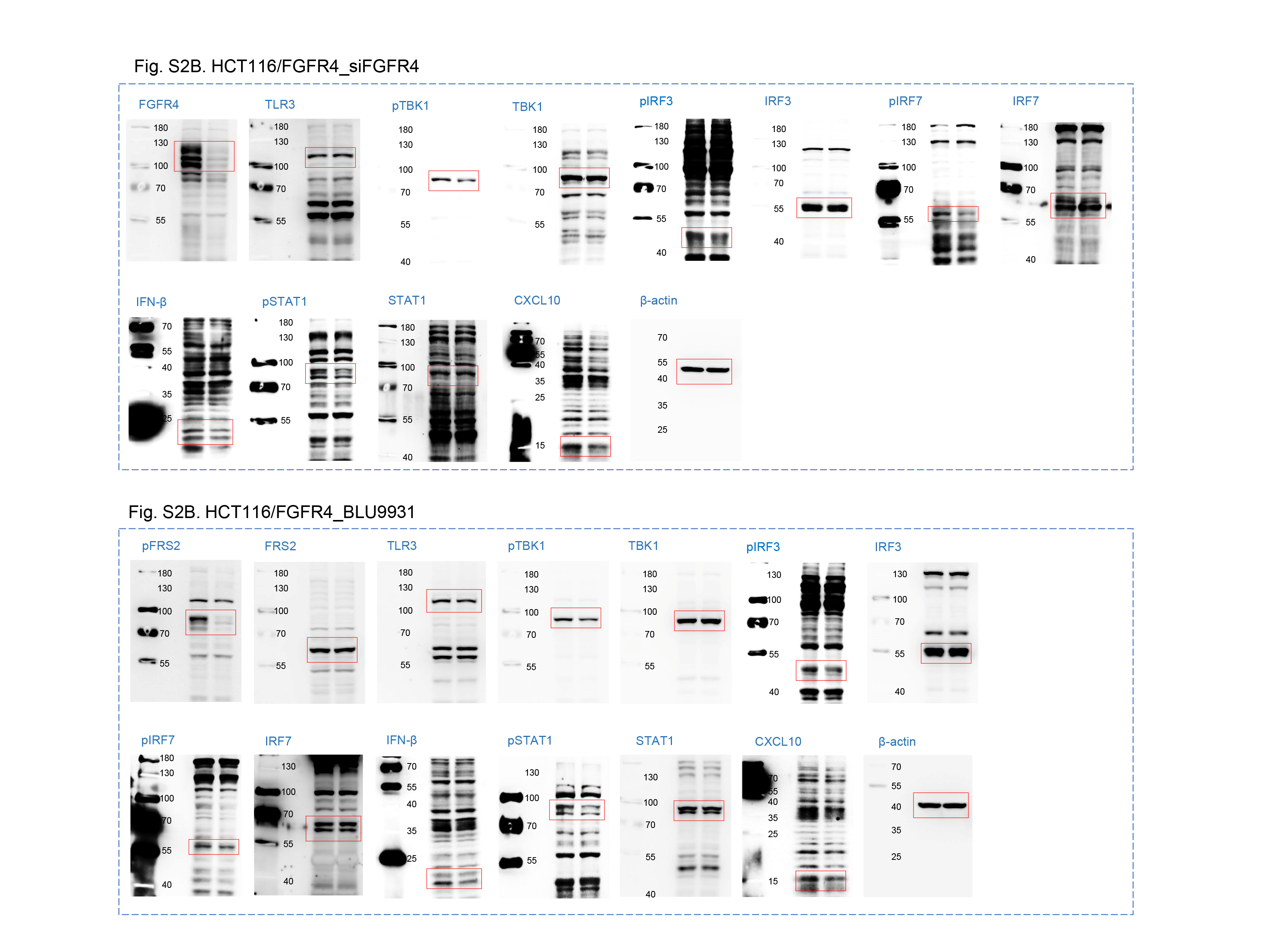

Supplement: Supplementary file 11 — Original WB figure-5 [file 41419_2025_7588_MOESM11_ESM.tif]

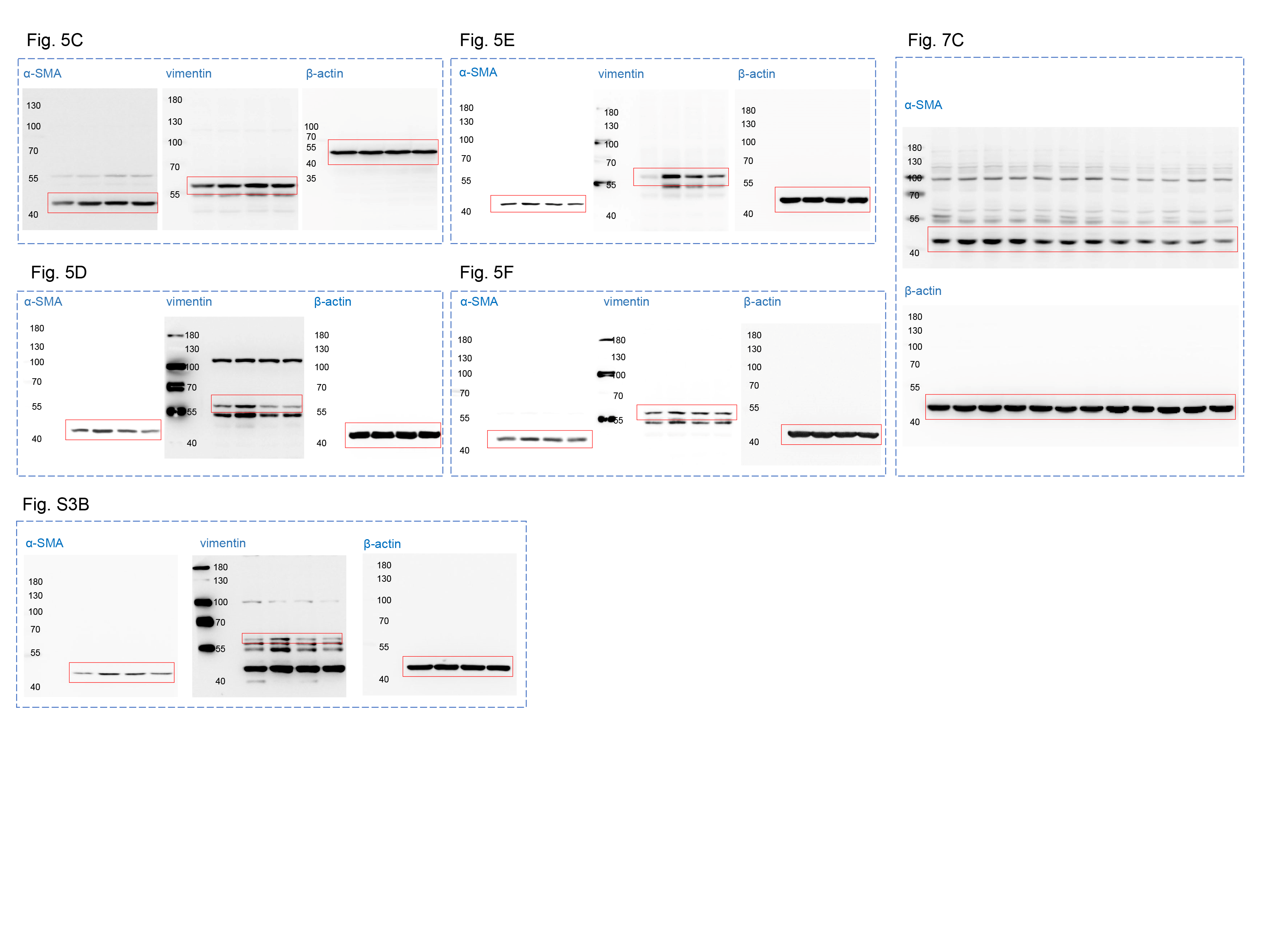

Supplement: Supplementary file 12 — Original WB figure-6 [file 41419_2025_7588_MOESM12_ESM.tif]
